# Nanomaterial-assisted pancreatic cancer theranostics

**DOI:** 10.1093/rb/rbaf054

**Published:** 2025-06-11

**Authors:** Yaqi Liu, Huachun Xu, Shihao Bai, Tianxiang Chen, Xuehua Ma, Jie Lin, Linglin Sun, Changyong Gao, Aiguo Wu, Qiang Li

**Affiliations:** College of Chemical Engineering, Zhejiang University of Technology, Hangzhou 310014, China; Ningbo Key Laboratory of Biomedical Imaging Probe Materials and Technology, Zhejiang International Cooperation Base of Biomedical Materials Technology and Application, Chinese Academy of Sciences (CAS) Key Laboratory of Magnetic Materials and Devices, Zhejiang Engineering Research Center for Biomedical Materials at Ningbo Cixi Institute of Biomedical Engineering, Institute of Biomedical Engineering at Ningbo Institute of Materials Technology and Engineering, Chinese Academy of Sciences, Ningbo 315201, China; Department of Radiology, The Affiliated People’s Hospital of Ningbo University, Ningbo 315100, China; Ningbo Key Laboratory of Biomedical Imaging Probe Materials and Technology, Zhejiang International Cooperation Base of Biomedical Materials Technology and Application, Chinese Academy of Sciences (CAS) Key Laboratory of Magnetic Materials and Devices, Zhejiang Engineering Research Center for Biomedical Materials at Ningbo Cixi Institute of Biomedical Engineering, Institute of Biomedical Engineering at Ningbo Institute of Materials Technology and Engineering, Chinese Academy of Sciences, Ningbo 315201, China; Ningbo Key Laboratory of Biomedical Imaging Probe Materials and Technology, Zhejiang International Cooperation Base of Biomedical Materials Technology and Application, Chinese Academy of Sciences (CAS) Key Laboratory of Magnetic Materials and Devices, Zhejiang Engineering Research Center for Biomedical Materials at Ningbo Cixi Institute of Biomedical Engineering, Institute of Biomedical Engineering at Ningbo Institute of Materials Technology and Engineering, Chinese Academy of Sciences, Ningbo 315201, China; Ningbo Key Laboratory of Biomedical Imaging Probe Materials and Technology, Zhejiang International Cooperation Base of Biomedical Materials Technology and Application, Chinese Academy of Sciences (CAS) Key Laboratory of Magnetic Materials and Devices, Zhejiang Engineering Research Center for Biomedical Materials at Ningbo Cixi Institute of Biomedical Engineering, Institute of Biomedical Engineering at Ningbo Institute of Materials Technology and Engineering, Chinese Academy of Sciences, Ningbo 315201, China; Ningbo Key Laboratory of Biomedical Imaging Probe Materials and Technology, Zhejiang International Cooperation Base of Biomedical Materials Technology and Application, Chinese Academy of Sciences (CAS) Key Laboratory of Magnetic Materials and Devices, Zhejiang Engineering Research Center for Biomedical Materials at Ningbo Cixi Institute of Biomedical Engineering, Institute of Biomedical Engineering at Ningbo Institute of Materials Technology and Engineering, Chinese Academy of Sciences, Ningbo 315201, China; Department of Radiology, The First Affiliated Hospital of Zhejiang Chinese Medical University (Zhejiang Provincial Hospital of Chinese Medicine), Hangzhou 310003, China; Ningbo Key Laboratory of Biomedical Imaging Probe Materials and Technology, Zhejiang International Cooperation Base of Biomedical Materials Technology and Application, Chinese Academy of Sciences (CAS) Key Laboratory of Magnetic Materials and Devices, Zhejiang Engineering Research Center for Biomedical Materials at Ningbo Cixi Institute of Biomedical Engineering, Institute of Biomedical Engineering at Ningbo Institute of Materials Technology and Engineering, Chinese Academy of Sciences, Ningbo 315201, China; Ningbo Key Laboratory of Biomedical Imaging Probe Materials and Technology, Zhejiang International Cooperation Base of Biomedical Materials Technology and Application, Chinese Academy of Sciences (CAS) Key Laboratory of Magnetic Materials and Devices, Zhejiang Engineering Research Center for Biomedical Materials at Ningbo Cixi Institute of Biomedical Engineering, Institute of Biomedical Engineering at Ningbo Institute of Materials Technology and Engineering, Chinese Academy of Sciences, Ningbo 315201, China; Department of Radiology, The Affiliated People’s Hospital of Ningbo University, Ningbo 315100, China; Department of Radiology, The First Affiliated Hospital of Zhejiang Chinese Medical University (Zhejiang Provincial Hospital of Chinese Medicine), Hangzhou 310003, China

**Keywords:** nanomaterials, boundaric imaging, therapeutic strategy, pancreatic cancer

## Abstract

Pancreatic cancer is one of the most lethal malignancies, largely due to the limitations of current imaging technologies and treatment strategies, which hinder early diagnosis and effective disease management. Achieving precise theranostics for pancreatic cancer has become a priority, and recent advances have focused on the development of novel nanomaterials with enhanced imaging capabilities and therapeutic functionalities. These nanomaterials, through surface modifications, can significantly improve the targeting and precision of both diagnostic and therapeutic applications. Recent progress in nanomaterial design has led to the creation of multifunctional platforms that not only enhance imaging but also improve therapeutic efficacy. These innovations have spurred significant interest in the application of nanomaterials for pancreatic cancer theranostics. In this review, we highlight recent developments in the use of nanomaterials for diagnostic imaging and precision therapy in pancreatic cancer. Various applications, including magnetic, optical, acoustic and radiological imaging, as well as therapeutic strategies such as chemodynamic therapy, light-based therapy, sonodynamic therapy and gene therapy, are discussed. Despite the promising potential of these nanomaterials, several challenges remain. These include optimizing targeting mechanisms, enhancing nanomaterial stability *in vivo*, overcoming biological barriers and ensuring safe and effective translation to clinical settings. Addressing these challenges will require further research and innovation. With sustained efforts, nanomaterial-assisted diagnostics and therapeutics have the potential to revolutionize the management of pancreatic cancer, ultimately improving early detection and treatment outcomes. Continued progress in this field could significantly enhance the overall prognosis for pancreatic cancer patients, making it a more treatable disease in the future.

## Graphical Abstract

**Figure:**
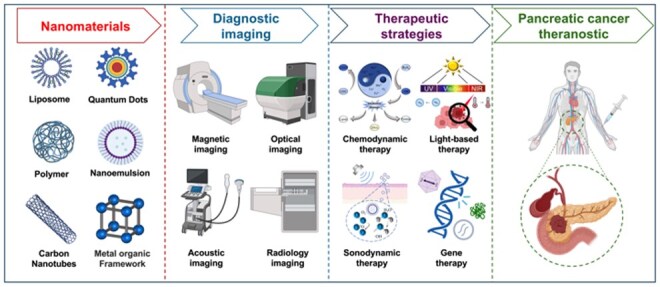

## Introduction

Pancreatic cancer remains one of the most lethal malignancies, with a five-year survival rate of only 10% and a global incidence that continues to rise [1–3]. The dismal survival rate is primarily attributed to delayed diagnosis, the inherently aggressive nature of the tumor, and the limited efficacy of current therapeutic approaches [4–6]. In its early stages, pancreatic cancer is typically asymptomatic and difficult to detect [7]. Furthermore, the deep anatomical location of the pancreas presents significant challenges for detection using conventional diagnostic technologies [8]. Common clinical imaging modalities, such as computed tomography (CT) and magnetic resonance imaging (MRI), are incapable of reliably detecting tumors smaller than 1 cm in diameter [9, 10]. Although endoscopic ultrasound (EUS) imaging can theoretically detect tumors as small as 2–3 mm in diameter, its effectiveness is highly dependent on operator expertise [11]. Thus, there is an urgent need to improve imaging techniques to enable accurate and early diagnosis of the boundaries of pancreatic cancer. In pancreatic cancer, accurate imaging of tumor boundaries is critical for early detection, precise surgical resection and targeted therapy, as the lesion margins represent transitional zones of molecular and cellular heterogeneity. Boundary imaging enables identification of subtle pathological changes at the tumor-normal interface, offering vital insights into tumor invasion, progression and therapeutic response-key elements for improving patient prognosis and advancing precision medicine [12].

Pancreatic cancer is an extremely aggressive malignancy, and its tumor microenvironment (TME) plays a crucial role in the development, progression and treatment resistance of the disease. The TME of pancreatic cancer is composed of a variety of components, including abundant cellular elements [such as cancer-associated fibroblasts (CAFs), immune cells, endothelial cells and nerve cells] and noncellular elements [such as the extracellular matrix (ECM)]. The interactions between these cellular and noncellular components form a complex ecosystem, providing a specialized niche for cancer cell growth and regulating tumor growth and invasive behavior [13, 14]. This complex microenvironment not only provides nutritional support for tumor cells but also promotes tumor immune evasion and treatment resistance through various mechanisms [15]. However, the complexity of the TME also poses challenges for research, particularly its spatial heterogeneity and the heterotypic interactions between cells. The emergence of heterogeneity not only affects cellular functions but can also lead to different disease progression trajectories, resulting in significant variations in the TME both among different patients and within the same tumor of a single patient [16]. In addition, the heterogeneity of the pancreatic cancer TME is not only present within the tumor but also extends to the tumor boundary. The components of the microenvironment at the tumor boundary, such as the ECM and CAFs, can form a physical barrier that restricts the infiltration of immune cells and the penetration of drugs. This barrier not only protects tumor cells from immune surveillance and drug attack but also promotes tumor invasion and metastasis [17].

Over the past few decades, various treatment modalities, including chemotherapy, radiotherapy and immunotherapy, have been explored to improve pancreatic cancer therapeutic effect [18–20]. However, the dense stromal microenvironment of pancreatic tumors presents a substantial barrier to effective drug delivery, further diminishing the efficacy of systemic treatments [21–23]. The disease is also characterized by a high propensity for metastasis and significant resistance to conventional therapies, including chemotherapy and radiotherapy. Clinical results have demonstrated that treatment regimens such as FOLFIRINOX/modified FOLFIRINOX (a combination of oxaliplatin, irinotecan, leucovorin and 5-fluorouracil) and gemcitabine (GEM) combined with albumin-bound paclitaxel (nab-paclitaxel) offer limited improvements in survival [24–27]. Consequently, the development of novel therapeutic strategies for pancreatic cancer is of critical importance.

**Figure 1.**
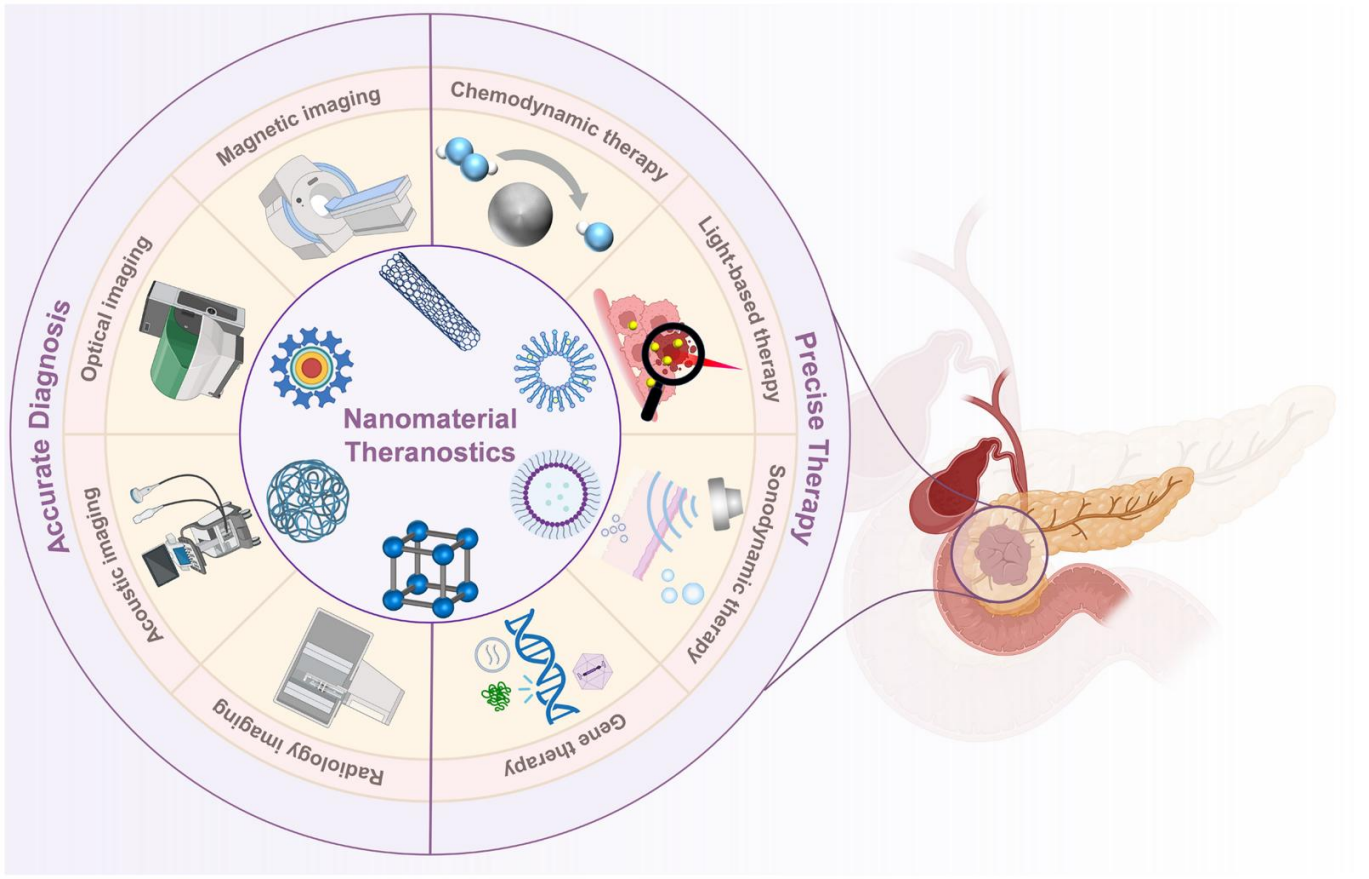
Schematic illustration of nanomaterial-assisted accurate diagnosis and precise therapy of pancreatic cancer.

To enhance the diagnostic and therapeutic efficacy of pancreatic cancer, various nanomaterials such as liposomes, micelles, mesoporous silica nanoparticles and iron oxide nanoparticles have been extensively investigated [28–34]. This progress report aims to advance the field of pancreatic cancer theranostics by summarizing and discussing recent developments in nanomaterial-assisted diagnostic and therapeutic approaches (Figure 1). First, advancements in nanomaterial-based diagnostic techniques, including optical, magnetic, computed tomography, positron emission tomography and multimodal imaging modalities, are reviewed. Subsequently, the applications of nanomaterials in the precision treatment of pancreatic cancer are highlighted. Finally, the challenges and prospects of nanomaterial-assisted imaging and therapy for pancreatic cancer are critically discussed.

## Nanomaterial-based diagnosis of pancreatic cancer

Pancreatic cancer is predominantly diagnosed at an advanced, metastatic stage, where it exhibits limited responsiveness to conventional therapeutic modalities, such as chemotherapy and radiotherapy. As a result, there is an urgent need for the development of more effective, noninvasive detection and imaging techniques that can facilitate the early diagnosis of pancreatic malignancies [35]. At present, a variety of imaging modalities, including CT, MRI, endoscopic techniques and ultrasound, are routinely employed in clinical practice for the early detection of pancreatic cancer. These approaches provide detailed and high-resolution images, thereby aiding clinicians in making accurate diagnoses and formulating appropriate treatment plans [36]. Simultaneously, the development of molecular imaging technologies incorporating specific probes or tracers has enhanced the ability to identify pancreatic cancer at its earliest stages [37]. These innovations offer novel tools that enable more precise and targeted diagnostic and therapeutic interventions [38, 39]. Despite these advancements, several challenges remain in achieving accurate and reliable diagnosis of pancreatic cancer. These challenges include the dense stromal architecture of pancreatic tumors, their relatively poor vascularization and the immunosuppressive microenvironment, all of which significantly impair the effective delivery of imaging agents to the tumor site [40].

To overcome these limitations, various forms of biological imaging nanomaterials have been developed for the diagnosis of pancreatic cancer. For example, the size of nanomaterials is comparable to that of biomolecules, enabling them to interact efficiently with biological systems. By surface-modifying specific targeting ligands (such as antibodies or peptides), nanoprobes can specifically bind to overexpressed markers on the surface of pancreatic cancer cells, such as epithelial cell adhesion molecule (EpCAM) or glycoprotein GPC-1 [41]. This specific binding not only improves the accuracy of diagnosis, but also enables noninvasive detection and real-time monitoring of tumors through techniques such as FI, MRI or PET [36]. In addition, nanomaterials can also be used to detect circulating tumor cells (CTCs) or circulating tumor DNA (ctDNA) in the blood. The detection of these liquid biopsy markers is of great significance for the early detection of pancreatic cancer. The high specific surface area and tunable surface properties of nanomaterials enable them to efficiently capture and enrich these rare markers, thereby improving the sensitivity and specificity of detection [4]. In the following sections, the recent progress in nanomaterials-assisted imaging strategies for pancreatic cancer will be discussed.

### Magnetic imaging

Magnetic imaging is a diagnostic technique that leverages the interaction between magnetic fields and magnetic materials to obtain detailed information about the internal structure of an object. Magnetic nanomaterials are capable of altering their magnetic state in response to an external magnetic field. This alteration can be detected using imaging modalities such as MRI and magnetic particle imaging (MPI). MRI uses magnetic fields and radiofrequency pulses to excite hydrogen nuclei in the body, generating images by detecting signals during their relaxation. Magnetic nanomaterials act as contrast agents that significantly shorten the relaxation time of the surrounding tissue, thereby enhancing the MRI signal. MPI, conversely, is an emerging imaging technique that directly detects the magnetic response of magnetic nanomaterials in a magnetic field. By detecting the magnetic response of these nanomaterials, the imaging signal can be significantly enhanced, thereby improving the contrast and resolution of the image and providing a clearer and more accurate diagnosis. Therefore, magnetic nanomaterials have a broad application prospect in magnetic imaging [42, 43].

Owing to its high spatial resolution and noninvasive characteristics, MRI serves as a noninvasive diagnostic tool in the clinical assessment of pancreatic cancer [44]. The enhancement of MRI capabilities by magnetic nanoparticles primarily results from their paramagnetic properties, which enable significant signal alterations under the influence of an external magnetic field, thereby improving both contrast and resolution [45]. The use of contrast agents further enhances the sensitivity and detection capabilities of MRI. Specifically, magnetic resonance contrast agents are classified into two categories based on their relaxation mechanisms: *T*_1_-weighted (longitudinal relaxation) contrast agents, which mainly include gadolinium (Gd^3+^) and manganese (Mn^2+^) nanoparticles that increase *T*_1_ relaxation times through paramagnetism, and *T*_2_-weighted (transverse relaxation) contrast agents, which mainly include SPIONs that generate dark signals by reducing *T*_2_ relaxation times [46]. These nanomaterials have shown significant advantages in the MRI diagnosis of pancreatic cancer. Magnetic nanomaterials can serve as MRI contrast agents, enhancing imaging contrast and resolution through their inherent paramagnetic properties. Moreover, magnetic nanomaterials can respond to internal stimuli (such as pH fluctuations or the activity of enzymes specific to the tumor microenvironment), thereby enabling the activation of the contrast agent and imaging within the tumor region, providing a comprehensive platform for the diagnosis of pancreatic cancer [47].

Gadolinium-based contrast agents (GBCA) have been widely employed in clinical practice to enhance the sensitivity of early pancreatic cancer diagnosis. Recent advancements focus on the development of targeted GBCAs, where gadolinium(III) chelates are conjugated with specific ligands designed to bind selectively to overexpressed biomarkers on pancreatic cancer cells. This targeted approach facilitates the preferential retention of biomolecule-bound GBCAs within tumor tissues, while unbound agents are rapidly cleared, thereby enhancing magnetic resonance imaging (MRI) contrast through differential accumulation and improving diagnostic precision [48]. Extradomain B fibronectin (EDB-FN), a component highly expressed in the fibrotic extracellular matrix of pancreatic cancer, constitutes a substantial proportion of the tumor volume. Leveraging this expression profile, gadolinium-based contrast agents (GBCAs) functionalized to target EDB-FN exhibit enhanced specificity and accumulation within pancreatic tumor tissues. This targeted approach enables significant signal amplification in MRI, offering a promising strategy for improving the diagnostic accuracy of pancreatic cancer [49]. The construction of modular self-assembling dendritic macromolecule nanosystems represents a promising strategy. By designing amphiphilic dendrimers containing multiple Gd³^+^ units, nano-micellar agents with exceptional relaxivity were developed for MRI applications in pancreatic cancer. The success of this imaging technology can be attributed to the high density of imaging units conjugated to the termini of the dendrimer on the nanoprobe surface, as well as the tumor accumulation and targeting mediated by the enhanced permeability and retention effect [50].

**Figure 2.**
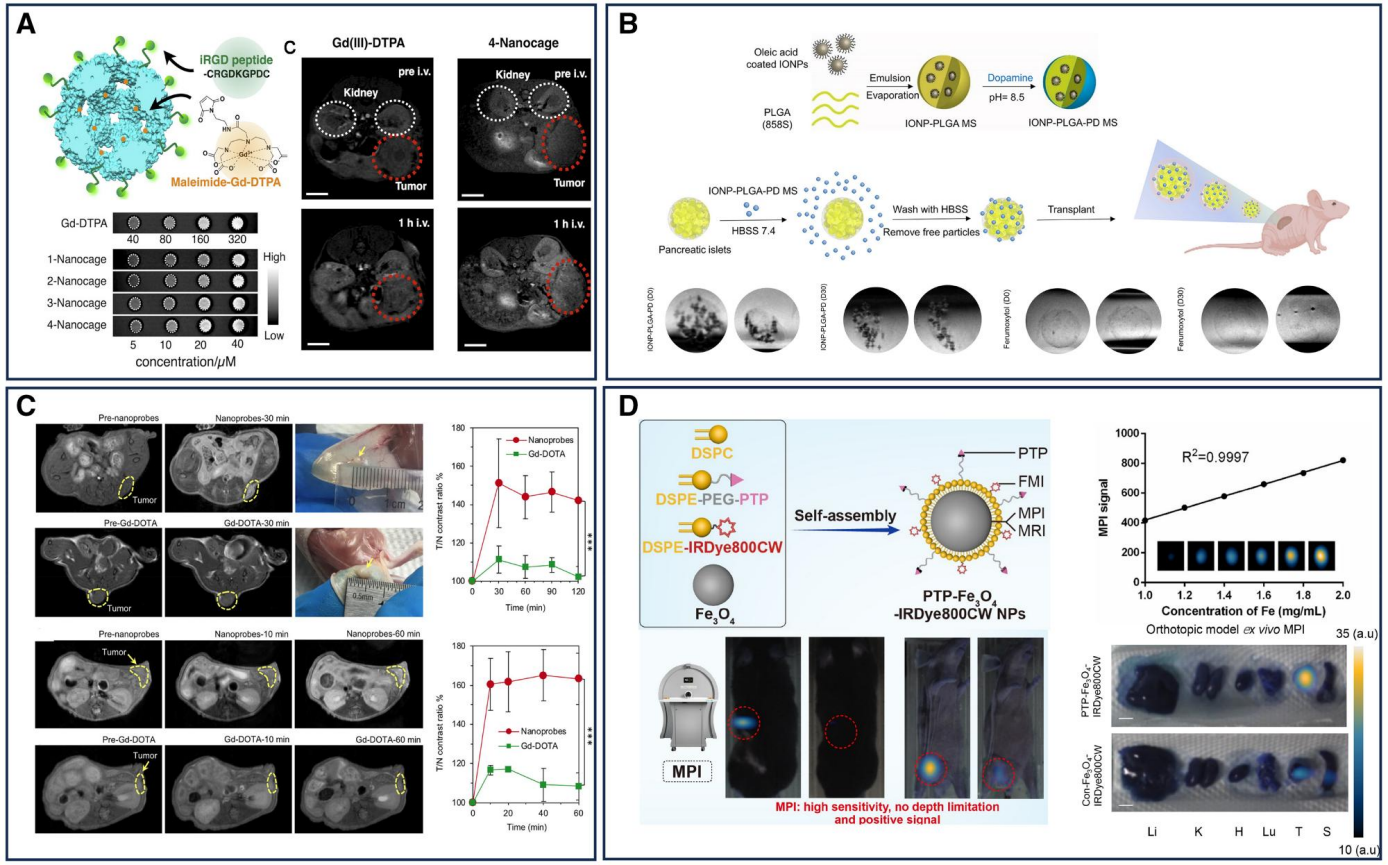
Nanomaterial-assisted magnetic imaging for the diagnosis of pancreatic cancer. (**A**) MRI of pancreatic tumors with Gd-based protein nanomaterials as targeted contrast agents. Reproduced with permission [53]. Copyright 2017, Elsevier. (**B**) The preparation of IONP-PLGA-PD MS and its application in islet labeling and tracking under MRI. Reproduced with permission [54]. Copyright 2023, Elsevier. (**C**) *In vivo* MRI of ultrasmall, orthotopic and spontaneous pancreatic tumors with MR-CA nanoprobes. Reproduced with permission [55]. Copyright 2021, American Chemical Society. (**D**) Superparamagnetic iron oxide nanomaterials for precise detection of pancreatic cancer in mice by MPI. Reproduced with permission [56]. Copyright 2022, Elsevier.

Additionally, MRI contrast agents can be improved in their pharmacokinetic properties and relaxivity by conjugation with macromolecular scaffolds, enabling better detection of cancer tissues. Specifically modified MRI nanoprobe accumulate in target cancer tissues by recognizing overexpressed receptors on cancer cells, and their sensitivity increases with the increased relaxivity of Gd(III) chelate contrast agents, which is related to the reduction of molecular tumbling [51]. Common scaffolds for conjugation, such as chemically synthesized polymer micelles, liposomes, inorganic nanoparticles and virus-like particles, have been extensively explored in preclinical studies. However, the majority of these investigations rely on nude mouse models, which fail to fully recapitulate the complex tumor microenvironment of human pancreatic cancer, particularly the dense stromal tissue characteristic of pancreatic tumors [52]. Genetically engineered tumor models, which more accurately mimic the pathogenesis of human tumors, have been utilized to evaluate a limited number of chemically conjugated MRI contrast agents. To address this gap, researchers have developed a nanomaterial based on small heat shock protein 16.5 (Hsp16.5), engineered to enhance the specificity and sensitivity of MRI for pancreatic cancer. This nanomaterial integrates a gadolinium (III) chelator with an iRGD peptide, demonstrating significantly improved *T_1_* relaxation rates compared to conventional contrast agents. To explore the impact of size on relaxivity, nanocages with varying numbers of hydrophobic domains (ranging from one to four) were designed. MRI data revealed that larger nanocages exhibited higher *T_1_* relaxivity, attributed to reduced molecular tumbling rates due to their increased size. The structural integrity of the nanocages, reinforced by hydrophobic domains, further enhanced relaxivity. In the Kras^G12D^; Trp53^R172H^; Pdx-1Cre (KPC) transgenic mouse model, the nanocages with four hydrophobic domains exhibited a tumor-to-normal contrast ratio of 115% at 60 min, markedly surpassing the performance of an equivalent dose of Gd-DTPA (Figure 2A) [53].

Although gadolinium-based nanomaterials have been widely investigated and utilized as MRI contrast agents in the clinical diagnosis of pancreatic cancer, their utility is constrained by a relatively short *in vivo* circulation lifetime and limited specificity, with reported specificity as low as 63% [57]. These limitations have spurred interest in developing Mn(II) complexes as alternatives to Gd(III) [58]. Manganese-based contrast agents, a prominent class of *T_1_* contrast agents, have garnered significant attention due to their responsiveness to glutathione (GSH) or pH, enabling valence changes and manganese release under acidic conditions [59]. However, the development of Mn(II)-based MRI contrast agents faces challenges in achieving high thermodynamic stability, kinetic inertness and relaxivity. Noncyclic ligand-based Mn(II) complexes often exhibit insufficient kinetic inertness due to weak metal–ligand binding, while macrocyclic ligand-based Mn(II) complexes, although highly stable and inert, are synthetically challenging due to their structural complexity. These factors highlight the need for innovative strategies to optimize manganese-based contrast agents for clinical applications. A team led by Prof. Mukherjee has developed a novel Zn(II)-responsive “smart” MRI contrast agent for pancreatic imaging, based on Mn(II)-complex-doped porous silica nanoparticles. By encapsulating the Mn(II) complex within porous silica nanoparticles and functionalizing the surface to achieve selective Zn(II) ion responsiveness, the researchers enhanced both the stability of the complex and its relaxivity. This was accomplished by restricting the molecular rotation of the complex, facilitating its specific accumulation in the pancreas and offering a promising tool for early pancreatic cancer diagnosis. The longitudinal relaxation rate (*r_1_*) of the contrast agent increased from 2.86 mM^−1^ s^−1^–13.27 mM^−1^ s^−1^ at a magnetic field strength of 1.41 T. In the presence of Zn (II) ions, the *r_1_* value further increased to 39.01 mM^−1^ s^−1^, demonstrating significant potential for improved imaging sensitivity [60].

In contrast to gadolinium- and manganese-based contrast agents, iron oxide nanoparticles are generally regarded as safer due to iron's role as an essential element in human physiology. SPIONs have gained FDA approval as MRI contrast agents, attributed to their low toxicity and well-characterized metabolic pathways. Functioning as negative contrast agents, SPIONs reduce signal intensity on *T*_2_-weighted images [61–63]. Numerous studies have validated the efficacy of SPIONs for *in vivo* pancreatic cancer imaging, with SPIONs-MRI having progressed through multiple clinical trials, highlighting its significant potential for clinical application in pancreatic cancer diagnostics [64]. Despite their advantages, the diagnostic sensitivity of iron oxide-based MRI contrast agents remains limited, largely due to background interference and the complex tumor microenvironment, which impedes efficient delivery via the EPR effect. To address these challenges, researchers have integrated nanomaterials with contrast agents to enhance imaging quality. For instance, embedding IONPs into a polymeric carrier enables consistent MRI intensity for tracking mesenchymal stem cells. A novel MRI contrast agent has been developed, comprising poly(lactic-co-glycolic acid) microparticles loaded with IONPs and coated with a tissue-adhesive polydopamine layer, specifically tailored for pancreatic islet imaging. This nanomaterial facilitates efficient and reliable islet labeling through a one-step process, improving cell adhesion while reducing nanoparticle degradation rates. Consequently, it enables long-term MRI visualization, producing stable MRI signals for up to 30 days, thus, offering a promising approach for improved pancreatic cancer imaging (Figure 2B) [54]. In recent years, antibodies have emerged as ideal targeting agents due to their ability to recognize specific cellular targets. Among these, the mucin 1 monoclonal antibody (MUC1) is notably overexpressed and aberrantly glycosylated in pancreatic cancer, where it plays a critical role in regulating cancer cell invasion and metastasis. By conjugating SPIONs with MUC1, researchers have achieved specific targeting of pancreatic cancer cells. These functionalized nanomaterials significantly reduced signal intensity in *T_2_*-weighted imaging across varying concentrations, with their magnetic resonance transverse relaxation rate demonstrating a strong linear correlation with iron concentration in solution, yielding correlation coefficients of 0.997, 0.980 and 0.983, respectively [65].

Probes are specialized molecules designed for detection and imaging, capable of binding to specific biological markers to monitor processes or disease states. Expanding on the capabilities of MRI contrast agents, MRI probes have advanced to not only enhance imaging contrast but also enable molecular imaging. These probes, incorporating specific targeting ligands and responsive functionalities, can precisely detect and visualize distinct molecules and biological processes, providing detailed molecular-level biological insights [66]. Professor Mi Peng and his team developed an MRI-based diagnostic strategy using contrast-enhancing nanoprobes (MR-CA nanoprobes) to detect tumor acidosis and monitor hypoxia in pancreatic tumors in real time. The pancreatic TME typically exhibits characteristics of hypoxia and acidity. The nanoprobe remains stable under normal physiological pH conditions (pH 7.4), but dissociates in the acidic tumor microenvironment (pH 6.5 or lower), releasing a large amount of manganese ions (Mn^2+^). These manganese ions can bind to proteins in the tumor microenvironment (such as albumin), significantly increasing the molecular relaxivity (*r_1_*) and thereby enhancing the MRI signal. In this way, the nanoprobe can specifically produce strong contrast enhancement in the acidic tumor regions, enabling high-sensitivity imaging of pancreatic tumors and allowing for the differentiation between hypoxic regions and normal regions within the tumor. This provides a new means for the early diagnosis of pancreatic cancer and the prediction of therapeutic efficacy. In a spontaneous pancreatic tumor model, the tumor-to-normal contrast ratio in mice treated with MR-CA nanoprobes reached 170%, significantly exceeding the less than 110% observed with Gd-DOTA as a control (Figure 2C) [55].

With the emergence of MPI, a novel imaging modality has been introduced. MPI leverages the collective magnetization properties of SPIONs, enabling direct, quantitative imaging with zero background signal, thereby producing high-contrast images comparable to nuclear medicine techniques [67]. Over the past decade, MPI has gained recognition as a complementary magnetic imaging tool to MRI, offering significant potential in cancer diagnosis, targeting and therapy. Its unique advantages, including high sensitivity, exceptional contrast and real-time monitoring capabilities, establish it as an innovative platform in oncology research and treatment. Notably, MPI’s ability to provide unlimited imaging depth makes it particularly suitable for visualizing deep-seated tumors, such as pancreatic cancer [68]. Xue *et al*. developed a targeted nanomaterial, PTP-Fe_3_O_4_-IRDye800CW, designed to specifically bind plectin-1, a protein highly expressed in pancreatic ductal adenocarcinoma cells, thereby enhancing imaging specificity and sensitivity. In MPI imaging, this nanomaterial demonstrated 85.72% ± 1.53% signal intensity two days postinjection, highlighting its potential for precise tumor visualization (Figure 2D) [56]. The integration of nanomaterials with MPI technology opens new avenues for advancing pancreatic cancer diagnosis and treatment. Continued optimization of nanomaterial design and MPI technology is expected to enable earlier detection, more accurate tumor localization and improved therapeutic monitoring, ultimately enhancing patient survival rates and quality of life.

MRI and MPI present significant advantages for pancreatic cancer diagnosis, including noninvasive imaging, exceptional soft-tissue contrast and the potential for highly targeted imaging through nanoparticle utilization [69]. However, several limitations hinder their clinical efficacy. While MRI exhibits high sensitivity, its ability to detect small pancreatic tumors, particularly those smaller than 1 cm in diameter during early disease stages, remains suboptimal. Additionally, the pancreas’s high mobility, influenced by its proximity to the stomach, intestines and other abdominal organs, complicates the acquisition of clear and precise MRI images. For MPI, despite its considerable promise, the technology remains experimental and has yet to achieve widespread clinical adoption. Key technical challenges, such as improving resolution, sensitivity and refining MPI equipment, must be resolved before its routine clinical application can be realized. Addressing these limitations is critical to unlocking the full diagnostic potential of these imaging modalities.

### Optical imaging

Compared to magnetic imaging, optical methods offer distinct advantages, including superior spatial resolution and real-time imaging capabilities. Pancreatic cancer is characterized by extensive regions of severe hypoxia, a key prognostic factor that influences tumor progression and therapeutic response. Optical imaging is particularly well-suited for detecting and mapping these hypoxic regions, providing critical insights into tumor aggressiveness, treatment efficacy and patient prognosis [23]. However, the application of optical imaging in pancreatic cancer is hindered by limited tissue penetration and reduced imaging depth due to light scattering and absorption [70]. Moreover, the TME of pancreatic cancer has unique pathological characteristics, such as acidic pH, hypoxia and overexpression of specific enzymes. Overcoming these challenges necessitates the development of novel imaging probes and advanced optical technologies. Nanomaterials can be designed to respond under these specific conditions, such as releasing fluorescent dyes in an acidic environment, activating photoacoustic signals under hypoxic conditions, or altering their physicochemical properties under the action of specific enzymes. This responsiveness enables nanomaterials to “turn on” or “enhance” signals within the complex TME, thereby further improving imaging outcomes [71]. Therefore, nanomaterials play a pivotal role in enhancing the depth, resolution and specificity of optical imaging, thereby improving both diagnostic accuracy and therapeutic monitoring. These innovations offer a promising avenue for the early detection and real-time assessment of pancreatic cancer, paving the way for more effective clinical interventions.

Fluorescence imaging, an optical technique that utilizes fluorescent molecules to emit visible light upon excitation, has transformed the visualization and interpretation of biological processes. This approach offers high sensitivity and specificity, making it particularly valuable for complex physiological environments and clinical applications [72]. Recent advances in molecular imaging have facilitated the development of fluorescence-guided surgical techniques for pancreatic cancer. Optimized nanoprobes enable precise labeling of pancreatic tumors, metastatic lymph nodes and distant lesions, providing strong contrast and clear differentiation from surrounding healthy tissues. Real-time fluorescent signals enhance surgical precision by delineating resection margins, guiding lymph node dissection and improving the detection of hepatic or peritoneal metastases that could influence surgical outcomes [73]. These nanoprobes are designed to selectively bind to molecular markers overexpressed on pancreatic cancer cells, including MUC1, MUC4, MUC5AC and MUC16 [74]. Their preferential accumulation in malignant tissues generates a strong fluorescence signal, facilitating accurate tumor visualization. In clinical studies, antibody-fluorophore conjugates targeting markers such as CEA, EGFR and VEGF-A have demonstrated effective intraoperative tumor imaging, underscoring the potential of fluorescence-guided surgery. While these approaches remain in early stages, emerging evidence supports their promise for enhancing pancreatic cancer resection [75]. Despite these advances, conventional optical imaging techniques are constrained by light attenuation and tissue autofluorescence, which limit imaging depth and signal clarity. Fluorescence detection in the near-infrared (NIR) spectrum addresses these challenges, offering reduced photon scattering, minimal tissue autofluorescence and improved penetration depth, thereby enhancing the signal-to-background ratio. This refined approach holds significant potential for improving the accuracy and efficacy of fluorescence-guided pancreatic cancer surgery [76]. Indocyanine green (ICG), a clinically approved near-infrared-I (NIR-I) fluorescent dye, has emerged as a valuable probe for image-guided pancreatic cancer surgery [77]. To enhance its fluorescence properties and photothermal conversion efficiency, Yan *et al*. designed supramolecular nanofibers (TP5-ICG NFs) by co-assembling thymosin pentapeptide (TP5) with ICG. The incorporation of TP5 facilitates hydrogen bonding interactions, modulating the self-assembly process and promoting the formation of long-range ordered fibrillar nanostructures that significantly amplify fluorescence signals. The development of such supramolecular nanomaterials, composed of water-soluble and clinically approved components, presents promising avenues for improving pancreatic cancer diagnostics and therapeutic interventions [78].

**Figure 3.**
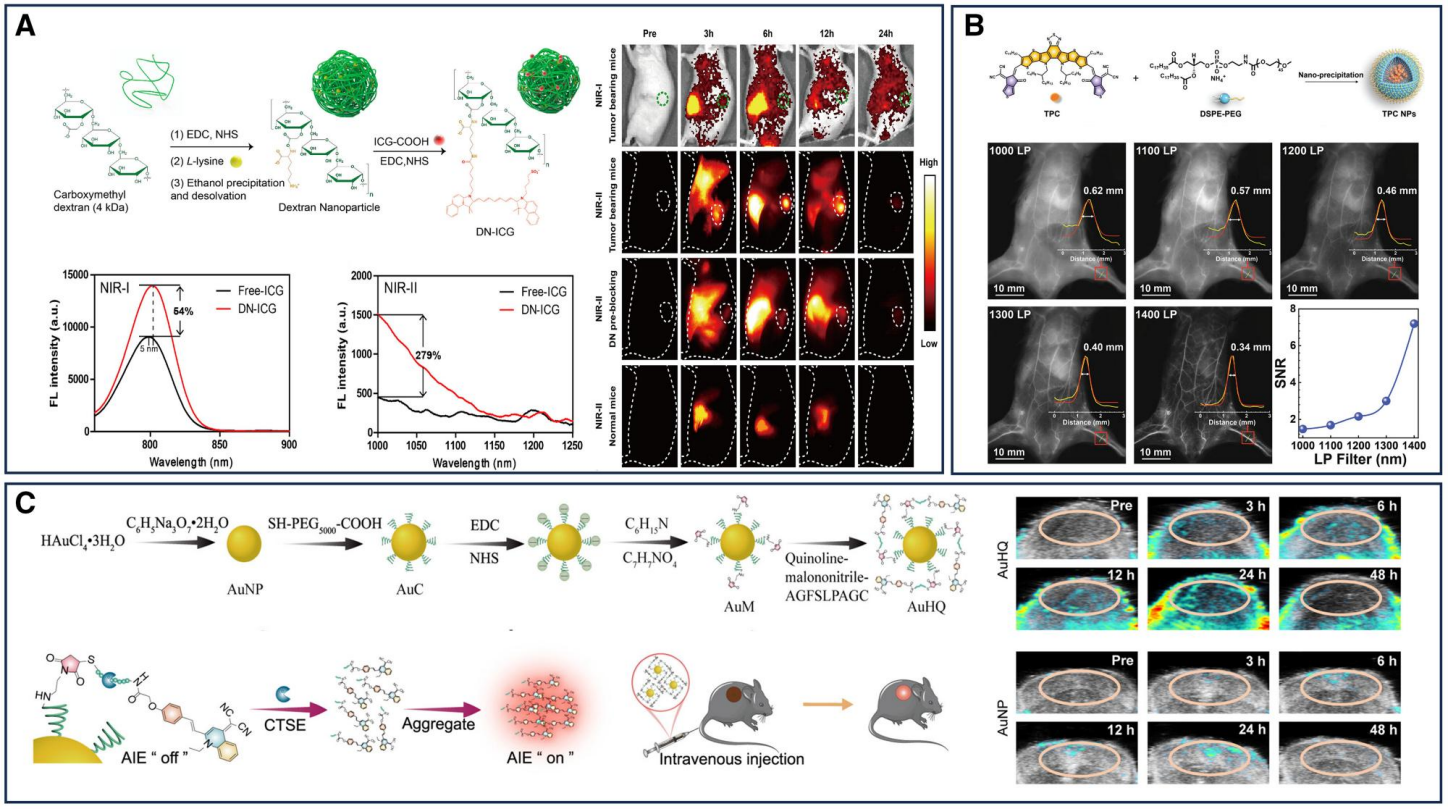
Nanomaterial-assisted optical imaging for the diagnosis of pancreatic cancer. (**A**) Highly biocompatible and biodegradable DN-ICG nanomaterials for near-Infrared-II imaging of deep pancreatic cancer tumors. Reproduced with permission [80]. Copyright 2021, American Chemical Society. (**B**) Versatile organic nanocarrier TPC for high-contrast targeted NIR-II photographs of pancreatic tumors. Reproduced with permission [81]. Copyright 2024, Wiley‐VCH GmbH. (**C**) AuHQ nanomaterials for precise dual self-assembly of AuNPs and AIE luminous in pancreatic tumors to facilitate fluorescence-photoacoustic imaging. Reproduced with permission [82]. Copyright 2024, American Chemical Society.

The near-infrared-II (NIR-II) spectral window, characterized by lower absorption and scattering in biological tissues and reduced autofluorescence compared to NIR-I, enables deeper tissue penetration and higher imaging contrast [79]. Building on these advantages, Prof. Sun and his team developed an ICG-conjugated dextran nanomaterial for dynamic imaging of tumor-associated macrophages (TAMs) in pancreatic cancer. By leveraging the specific binding affinity of dextran for SIGN-R1 receptors on the surface of TAMs, this nanomaterial circumvents the challenge of nonspecific nanoprobe interactions with cancer cells while enhancing the NIR-II fluorescence performance of ICG. The resulting nanoprobe exhibited a 279% increase in fluorescence intensity in the NIR-II window compared to free ICG, demonstrating deep-tissue imaging capabilities with a high signal-to-background ratio (SBR = 7) in a murine model. Furthermore, its favorable *in vivo* metabolizability reduced background fluorescence, further improving imaging precision and quality (Figure 3A) [80]. Beyond the clinically approved indocyanine green (ICG), a new generation of near-infrared-II (NIR-II) fluorescent probes has been developed to address diverse biomedical imaging needs. These include single-walled carbon nanotubes, quantum dots and semiconducting polymer nanoparticles, which exhibit exceptional optical properties and can respond dynamically to specific molecular or environmental changes [83–86]. However, many NIR-II fluorophores suffer from inherently low brightness, attributed to several factors: only a fraction of their emission spectrum falls within the NIR-II region, absorption wavelengths may not align with commonly used excitation sources, and enhancements in quantum yield (QY) often coincide with a blue shift that reduces extinction coefficients at 808 nm, thereby diminishing NIR-II emission. Effective probe design requires optimization of multiple parameters, including QY, extinction coefficient and spectral emission characteristics [87].

To address these limitations, Huang *et al*. developed a semiconductor polymer nanoparticle (L1057 NPs) system for NIR-II fluorescence imaging to enable dynamic monitoring of pancreatic cancer. With an emission spectrum predominantly confined to the NIR-II region, the material exhibited a high extinction coefficient and a quantum yield of 1.25%, generating strong NIR-II fluorescence under 980 nm laser excitation. This facilitated clear visualization of tumors and their surrounding vasculature, offering a promising platform for early pancreatic cancer detection and intervention [88]. Despite the advantages of NIR fluorescent dyes in tissue penetration, materials such as semiconducting polymers may experience significant signal attenuation in deep-seated tumors, particularly within the pancreatic microenvironment. In recent years, organic small molecules have emerged as promising candidates for NIR-II fluorophore development, as precise electronic structure tuning enables red-shifting of emission wavelengths from the visible to the NIR region. This shift is critical for reducing tissue autofluorescence, thereby improving imaging contrast and depth [89, 90]. By incorporating donor (D) and acceptor (A) structures, the π-conjugation length can be extended, resulting in a narrowed bandgap and red-shifted absorption profiles. Li *et al*. developed an A-D-A-type conjugated small-molecule nanomaterial (TPC) optimized for high-contrast targeted bioimaging of pancreatic cancer in the NIR-II region. To enhance tumor-targeting capability, the researchers camouflaged TPC NPs with pancreatic cancer cell membranes (forming TPC CNPs), enabling them to specifically recognize and target pancreatic tumor cells. This camouflage strategy leverages the specific molecular markers on the tumor cell membranes, which can interact with cells or the extracellular matrix in the tumor microenvironment, thereby increasing the accumulation and retention of the nanomaterials at the tumor site. The design overcame the quantum yield limitations imposed by the energy gap law, achieving bright NIR-II fluorescence with a quantum yield of 9.8%. *In vivo* imaging using a 1400 nm long-pass filter yielded a signal-to-noise ratio of 7.19, highlighting the nanomaterial’s potential for high-resolution deep-tissue imaging (Figure 3B) [81].

Conventional photosensitizers often suffer from fluorescence quenching due to π-π stacking in aggregated states, limiting their *in vivo* applications. In contrast, aggregation-induced emission (AIE) materials exhibit a large Stokes shift and superior photostability, making them highly advantageous for imaging and diagnostics [91]. AIE, first introduced by Ben Zhong Tang’s research team in 2001, describes a phenomenon in which molecular aggregation enhances fluorescence intensity. This unique property has driven the development of ultra-bright fluorescent nanomaterials for biomedical applications [92]. Weihong Zhu’s team introduced an enzyme-activatable AIE probe, QM-HSP-CPP, for intraoperative pathological fluorescence diagnosis of pancreatic cancer. By integrating the AIE core QM-COOH, enzyme-responsive peptide HSP and water-soluble transmembrane peptide CPP, the probe improved target specificity and membrane penetration efficiency, thereby enhancing imaging resolution. Experimental data demonstrated that QM-HSP exhibited classical AIE behavior in DMSO-water mixtures, with fluorescence intensity markedly increasing as the water fraction rose from 40% to 98%. Furthermore, its low tissue autofluorescence and strong penetration capability enabled real-time intraoperative pathological diagnosis of pancreatic cancer tissue sections, addressing the limitations of conventional histological staining techniques such as hematoxylin-eosin and immunohistochemistry [93].

Fluorescence imaging, when integrated with minimally invasive surgical tools, enables precise tumor localization, including the detection of microscopic lesions that are otherwise challenging to identify. Song *et al*. developed an organic small-molecule-based nanomaterial, BTz-IC, for pancreatic cancer diagnosis via NIR-II fluorescence imaging. The molecular design incorporated electron-accepting groups to facilitate fluorescence emission in the NIR-II region under 808 nm laser excitation. Under minimally invasive endoscopic guidance, the nanomaterial significantly improved the detection of deep-seated pancreatic tumors. *In vivo* experiments demonstrated that BTz-IC nanoparticles exhibited increasing fluorescence signals with rising concentration, reaching a maximum signal-to-background ratio (SBR) of 2.88 at 4 h postinjection, highlighting its potential for enhanced deep-tissue imaging [94].

While fluorescence imaging is a powerful tool in biomedical research, its effectiveness is often constrained by light scattering and absorption, particularly in deep tissue imaging. Photoacoustic (PA) imaging has emerged as a hybrid modality that overcomes these limitations by integrating the high spatial resolution of optical imaging with the deep tissue penetration of ultrasound. This technique leverages the photoacoustic effect, wherein short pulses of laser light are absorbed by tissues, inducing localized thermal expansion and the subsequent emission of ultrasound waves. These acoustic signals are then detected and reconstructed into high-resolution images, enabling detailed visualization of tissue structures. By providing a noninvasive, real-time imaging platform that directly reflects optical absorption properties, PA imaging offers a promising approach for deep-tissue imaging and functional analysis in biomedical applications [95].

Despite its advantages, PA imaging still faces several challenges in pancreatic cancer diagnosis, including a low signal-to-noise ratio, insufficient image contrast and limited imaging depth. These limitations hinder its sensitivity and specificity, particularly for the detection of deep-seated tumors such as pancreatic cancer [96]. To address these issues, researchers have explored novel strategies, including the use of 1,2-distearoyl-sn-glycero-3-phosphoethanolamine-polyethylene glycol (DSPE-PEG) nanomaterials to encapsulate PA probes, such as octapyrrolyl porphyrin. This approach overcomes the poor target specificity of conventional PA probes by integrating a conjugated porphyrin (CP) with a high extinction coefficient and a cyclic RGD (cRGD) peptide, an αvβ3 receptor ligand. This design enhances cell membrane penetration efficiency, thereby improving the resolution of PA imaging. Using a circular array PA tomography system, peak accumulation of the probe in pancreatic tumors was observed at 9 h postinjection. Histological analysis further confirmed clear delineation of tumor tissue from surrounding normal tissue in both subcutaneous and *in situ* pancreatic cancer models [97]. Gold nanomaterials have also shown significant potential for PA imaging in oncology, owing to their excellent biocompatibility and capacity for multifunctional modifications [98]. Fan *et al*. developed a bimodal fluorescence-luminescence-photoacoustic imaging system using an AIE molecule conjugated to gold nanoparticles (AuNPs) (AuHQ) for imaging-guided photothermal immunotherapy of pancreatic cancer. The AIE molecules and AuNPs were linked via peptide chains that are cleaved by the CTSE enzyme, inducing the self-assembly of large aggregates. This process markedly enhanced PA signal intensity, thereby improving both the sensitivity and specificity of imaging. After 24 h of incubation with CTSE, the PA signal of the AuHQ nanomaterial was fivefold greater than that of untreated AuNPs, demonstrating its potential for highly sensitive imaging and therapeutic monitoring (Figure 3C) [82].

### Acoustic imaging

Ultrasound, as a mechanical wave-based imaging modality, is widely regarded as a safe diagnostic tool due to its absence of ionizing radiation. It enables real-time dynamic assessment of both organ function and hemodynamics while simultaneously detecting pathological changes. Moreover, its capacity for real-time, cross-sectional imaging from multiple orientations allows for the concurrent acquisition of both functional and morphological information, significantly enhancing the detection and characterization of lesions. This dual capability makes ultrasound an indispensable tool in clinical imaging [99]. Endoscopic ultrasound (EUS) is particularly valuable for the early detection of pancreatic cancer due to its high sensitivity. The retroperitoneal location of the pancreas presents a challenge for conventional imaging techniques, such as transabdominal ultrasound, which can be affected by gastrointestinal gas and abdominal wall adiposity, limiting visualization-particularly of the pancreatic tail. By integrating endoscopy with ultrasound technology, EUS facilitates high-resolution, multi-axis imaging in close proximity to the pancreas, mitigating these limitations. The diagnostic performance of EUS varies according to tumor stage, with reported sensitivities and specificities of 72% and 90% for T1-T2 stage tumors and 90% and 72% for T3-T4 stage tumors, respectively. Furthermore, EUS is capable of detecting pancreatic lesions as small as 2–3 mm and is routinely employed for fine-needle aspiration (FNA) or biopsy in patients with suspected malignancies, providing crucial histopathological confirmation [11].

Despite its advantages, conventional endoscopic ultrasound (EUS) has limitations in differentiating pancreatic cancer from benign pancreatic lesions, particularly as many malignant tumors exhibit a cachectic morphology. To improve diagnostic accuracy, additional imaging parameters, such as tumor size and margin regularity, have been investigated, with combined diagnostic approaches demonstrating enhanced sensitivity and specificity [4]. Recent advancements in contrast-enhanced endoscopic ultrasonography (CE-EUS) utilizing nanomaterials have further expanded the capabilities of EUS, offering a minimally invasive method with superior sensitivity and diagnostic accuracy compared to conventional techniques. Meta-analyses have confirmed the high sensitivity and specificity of CEH EUS in pancreatic cancer detection, underscoring its potential to refine current diagnostic strategies and improve early detection in clinical practice [100].

Beyond EUS, ultrasonography remains a pivotal tool in the diagnosis of pancreatic cancer. By employing high-frequency sound waves to generate real-time images of internal structures, this technique offers several advantages in clinical practice, including cost-effectiveness, nonionizing radiation and deep tissue penetration [101]. Recent advances in ultrasound contrast agents have further expanded its diagnostic utility, particularly through the development of microbubbles. Microbubbles, typically composed of perfluorocarbon cores encapsulated within stabilizing shells of phospholipids, polymers or proteins, exhibit strong backscattering properties when exposed to ultrasound waves. Their ability to generate harmonic frequencies upon insonation significantly enhances image contrast, enabling more precise visualization of tissue architecture and vascularization [102]. Beyond improving imaging resolution, nano-microbubble technology has facilitated the convergence of molecular imaging and targeted therapy. Functionalization of microbubble surfaces with targeting ligands allows for selective binding to tumor-specific biomarkers, enabling precise, real-time imaging and localized therapeutic delivery. In addition, nanomaterials can also be used to enhance the ablation effect of high-intensity focused ultrasound (HIFU) by encapsulating substances such as perfluorohexane (PFH) and realizing liquid–gas phase transition under ultrasound radiation, thus, further improving the resolution and diagnostic capability of ultrasound imaging. The integration of microbubbles with nanomaterials, driven by advances in nanotechnology and materials science, has further refined ultrasound-based diagnostics and therapeutics, positioning ultrasound as a more precise and versatile tool in the management of pancreatic cancer.

**Figure 4.**
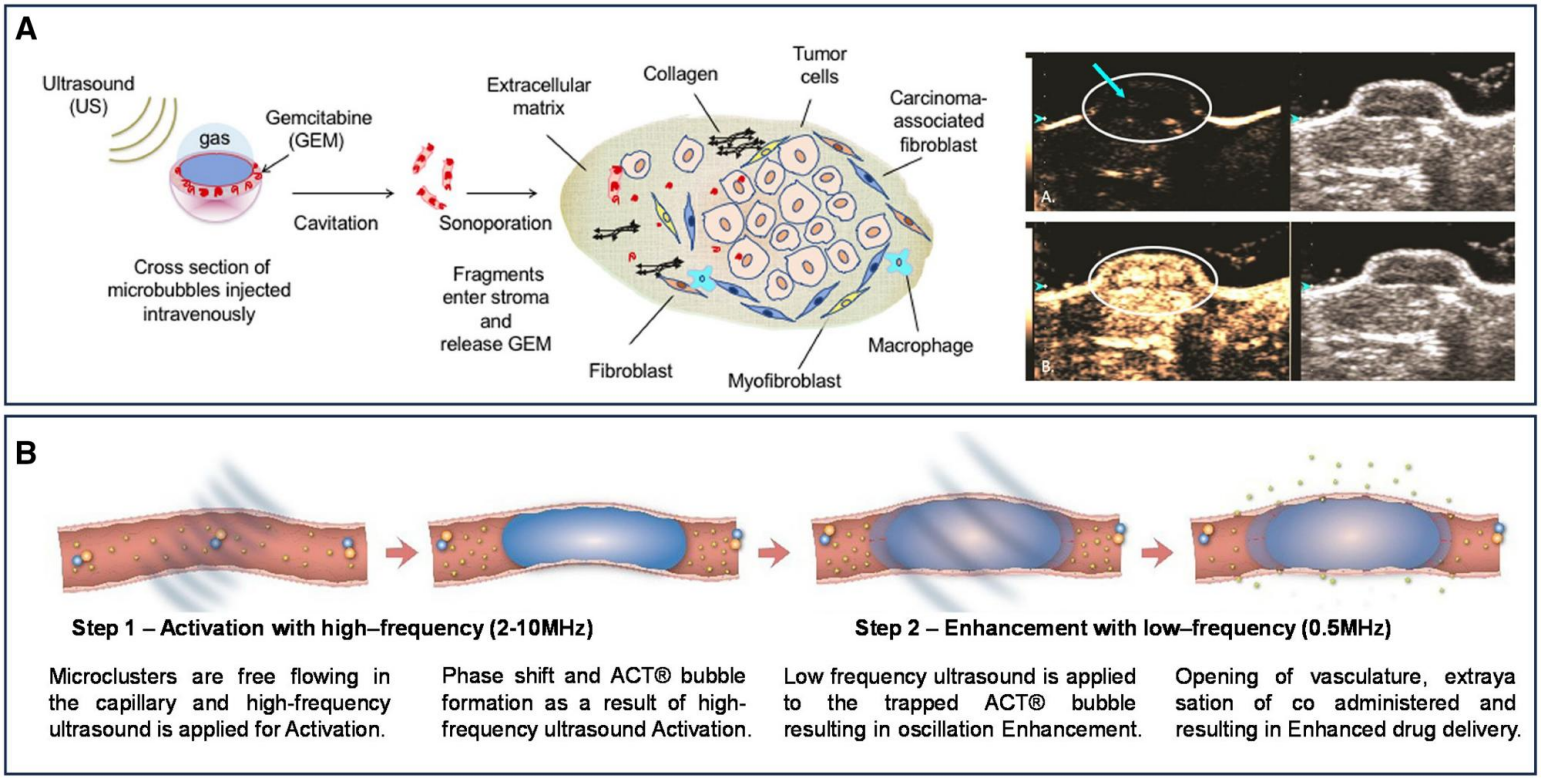
Nanomaterial-assisted acoustic imaging for the diagnosis of pancreatic cancer. (**A**) Ultrasound-triggered delivery of gemcitabine to pancreatic ductal adenocarcinoma stroma via drug-loaded ultrasound contrast microbubbles. Reproduced with permission [106]. Copyright 2021, Elsevier. (**B**) The ACT^®^ mechanism of ultrasound-activated microbubbles for enhanced tumor drug delivery. Reproduced with permission [107]. Copyright 2022, Elsevier.

Ultrasound contrast agents (UCAs) typically are composed of a gaseous core encapsulated by a stabilizing shell, significantly enhancing the reflection of ultrasound waves from biological tissues and blood. This signal amplification improves both the sensitivity and specificity of lesion detection. Upon insonation, UCAs undergo cavitation effects, including oscillation and rupture, which generate localized temperature and pressure fluctuations, further enhancing image resolution and contrast [103]. Although pancreatic cancer is typically hypovascular, its perfusion is sufficient to permit the infiltration of microbubbles, enabling effective contrast-enhanced ultrasound imaging. Recent studies have demonstrated that the combination of diagnostic ultrasound and UCAs can extend the therapeutic window, thereby improving clinical outcomes in pancreatic cancer management. The selection of targeting ligands is a critical factor in UCA design [104]. For instance, microbubbles conjugated with a single-chain antibody (Thy1-scFv), which selectively binds to the Thy1 antigen in human and murine pancreatic cancer, enable targeted ultrasound imaging with improved specificity [105]. Beyond diagnostic applications, ultrasound-based imaging platforms can be leveraged for therapeutic delivery. Drugs can be encapsulated within microbubbles or co-administered with contrast agents, facilitating ultrasound-triggered drug release at the target site. A poly(lactide) (PLA)-coated microbubble system has been developed for pancreatic cancer therapy, encapsulating gemcitabine while preserving both ultrasound contrast activity and drug efficacy. Ultrasound-mediated cavitation enhances drug permeability, promoting localized drug delivery and improving therapeutic outcomes (Figure 4A) [106]. Larger UCA microbubbles demonstrate superior vascular penetration and extravascular diffusion, further expanding the potential of ultrasound-based strategies. Acoustic Cluster Therapy (ACT^®^) is an advanced microbubble platform that employs high-frequency ultrasound to convert microdroplets into vaporized microbubbles (20–25 μm in diameter). These enlarged bubbles occupy a greater vascular volume and exhibit prolonged intravascular retention, significantly enhancing ultrasound imaging contrast and facilitating more effective therapeutic intervention (Figure 4B) [107].

### Computed tomography imaging

CT imaging is a widely employed diagnostic modality, valued for its cost-effectiveness and comprehensive anatomical coverage, making it an essential tool in clinical practice. Its ability to generate high-resolution images of tissues and organs has significantly advanced diagnostic medicine, particularly in the detection and evaluation of malignancies [108]. In pancreatic cancer diagnosis, CT imaging demonstrates a sensitivity of 76% to 92% and a specificity of approximately 67% [11]. However, a major limitation of CT lies in its reduced capacity to differentiate subtle soft tissue changes, largely due to the similar mass attenuation coefficients of pancreatic tumors and the surrounding pancreatic parenchyma. Furthermore, the dense desmoplastic stroma and hypervascular nature of pancreatic cancer often result in the formation of characteristic low-density masses, complicating accurate tumor delineation. These inherent challenges underscore the need for complementary imaging techniques or contrast-enhanced strategies to improve diagnostic accuracy in pancreatic cancer detection and staging [109].

**Figure 5.**
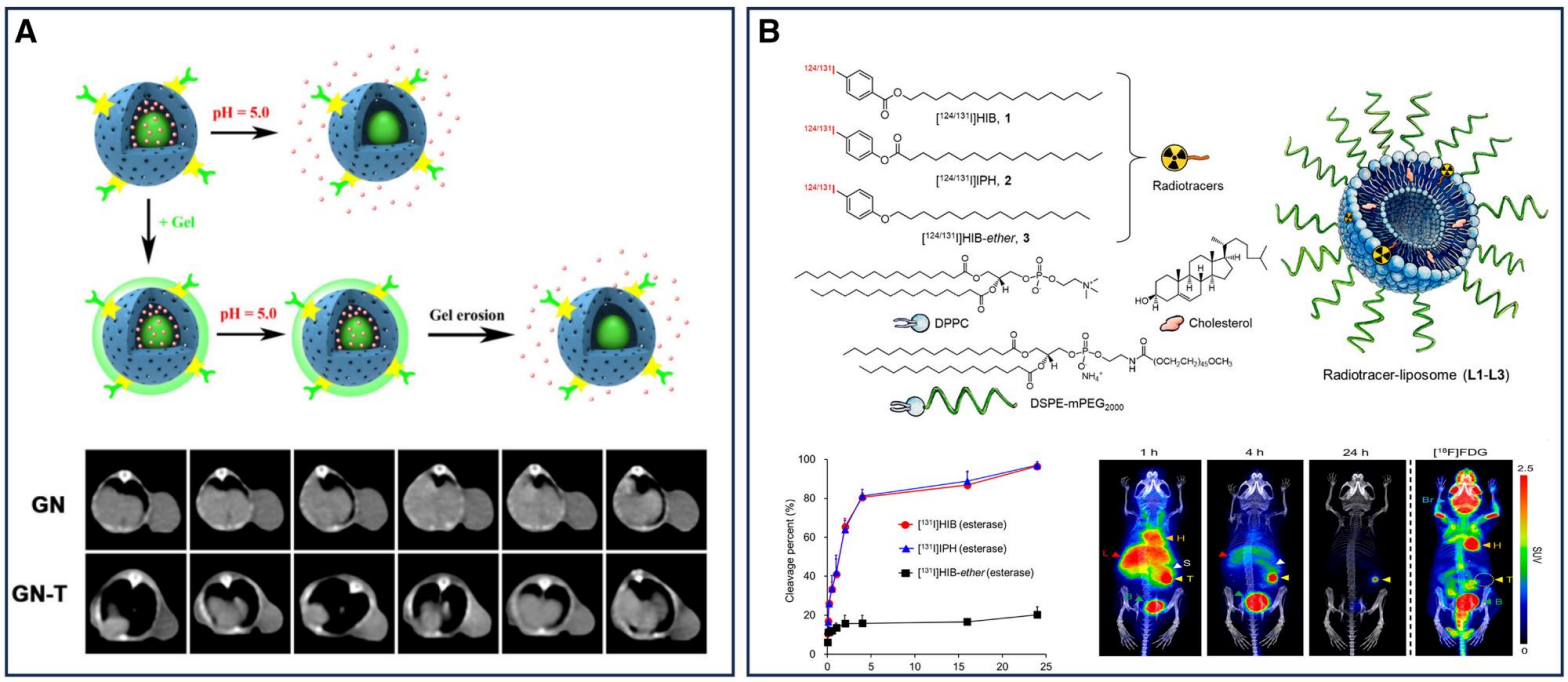
Nanomaterial-assisted radiology imaging for the diagnosis of pancreatic cancer. (**A**) Thermosensitive gel specifically accumulates in PDX and enhances CT imaging. Reproduced with permission [110]. Copyright 2020, Elsevier. (**B**) HIB liposomes enable high-contrast imaging of pancreatic cancer through differences in esterase activity between tumor and normal organs. Reproduced with permission [111]. Copyright 2021, American Chemical Society.

To overcome the limitations of conventional CT imaging, exogenous contrast agents with enhanced X-ray attenuation properties have been developed to improve tissue differentiation. Among these, nanomaterial-based contrast agents have demonstrated significant potential in enhancing both imaging sensitivity and contrast. Metal-based inorganic nanoparticles, in particular, have attracted considerable interest as CT contrast agents due to their high atomic number and large X-ray attenuation coefficients (Figure 5A) [110]. These nanomaterials can be surface-engineered to improve biocompatibility and stability while being tailored with specific shapes and functionalities to facilitate targeted accumulation within tumor regions. This localized enrichment enhances CT signal intensity, thereby improving the precision of both pancreatic cancer diagnosis and treatment planning [112].

### Positron emission tomography imaging

Positron emission tomography (PET) is a powerful imaging modality in nuclear medicine that enables real-time visualization of biological processes *in vivo* through the detection of radiotracers labeled with positron-emitting isotopes [113]. By integrating metabolic imaging with anatomical modalities such as CT or MRI, PET enhances the characterization of tumors, providing precise localization and improved differentiation of pancreatic cancer. Pancreatic cancer is highly infiltrative and prone to early metastasis, necessitating whole-body imaging for comprehensive disease assessment. PET enables the simultaneous detection of both primary pancreatic tumors and distant metastatic lesions, offering a more complete evaluation of disease progression. In addition to its high sensitivity in detecting small pancreatic lesions, PET demonstrates strong specificity in distinguishing between benign and malignant pancreatic masses [114]. One of the most widely used radiotracers in PET imaging is fluorodeoxyglucose labeled with fluorine-18 (^18^F-FDG), which plays a crucial role in cancer staging, treatment response assessment and recurrence monitoring. The diagnostic utility of ^18^F-FDG PET in pancreatic cancer is largely attributed to KRAS-driven metabolic reprogramming, which increases glucose uptake in tumor cells. This enhanced metabolic activity facilitates the selective accumulation of ^18^F-FDG, enabling precise tumor visualization and improving the accuracy of pancreatic cancer diagnosis [115].

The application of PET imaging in pancreatic cancer is challenged by the pancreas’s deep anatomical location within the abdomen, surrounded by large organs that can interfere with the efficient delivery of PET tracers such as ^18^F-FDG. These factors limit tracer accumulation in the tumor and hinder the acquisition of high-contrast images [36]. To address this limitation, nanomaterial-based PET imaging strategies have been developed to improve the early detection of pancreatic cancer. One approach involves encapsulating esterase-sensitive radiotracers within liposomes, enabling prolonged tumor retention while facilitating rapid clearance from the liver and spleen. This strategy significantly enhances PET imaging contrast, improving lesion detection and delineation. Further modifications to the liposomal surface, such as folate conjugation, enhance tumor targeting, enabling the visualization of tumors as small as a few millimeters within 4 h of imaging. By 24 h postinjection, more than 9% of total radioactivity localizes within the tumor, further increasing the sensitivity and accuracy of PET imaging (Figure 5B) [111]. The use of copper-64 (^64^Cu), owing to its longer half-life, allows for delayed PET imaging, thereby enhancing tumor uptake and image contrast. ^64^Cu-labeled factor VII with an inhibited active site (FVIIai) has been employed to detect tissue factor (TF) expression in pancreatic cancer. ^64^Cu-NOTA-FVIIai PET imaging enables specific identification of TF-positive tumors, with increasing tumor-to-background contrast over time, providing a promising noninvasive approach for pancreatic cancer diagnosis and treatment monitoring [116]. In addition to the aforementioned advantages, ^64^Cu also exhibits many significant benefits as a PET imaging radionuclide. During its decay process, the positrons released by ^64^Cu interact with electrons to undergo annihilation reactions, producing gamma photons. These gamma photons can be precisely detected by PET detectors and are used in image reconstruction techniques to achieve the visualization of tumors [117]. The radiolabeling technology of ^64^Cu is relatively mature and can be combined with a variety of biomolecules (such as antibodies and peptides) to form targeted PET imaging probes. These probes can specifically bind to certain receptors or proteins on the surface of pancreatic cancer cells (such as PDGFRβ and CLDN4), thereby achieving precise imaging of pancreatic cancer [118]. Yang *et al*. demonstrated the potential of Trop2 as a therapeutic target for pancreatic cancer through their research on the PET imaging and radioimmunotherapy (RIT) applications of ^64^Cu/^177^Lu-labeled anti-Trop2 monoclonal antibody (hIMB1636) in pancreatic cancer tumor models. However, in addition to Trop2, other molecular targets are also worth further investigation to expand the PET imaging and therapeutic strategies for pancreatic cancer [119]. Professor Katherine W. Ferrara identified Claudin-4 (CLDN4) as a PET imaging target for pancreatic through spatial transcriptomics and proteomics analyses. The study utilized a ^64^Cu-labeled peptide probe (^64^Cu-C4BP) targeting CLDN4 to achieve high-specificity imaging of pancreatic cancer tumors and metastases. This peptide-based PET imaging method not only allows for noninvasive assessment of CLDN4 expression levels but also enables high-sensitivity tumor detection in mouse models. This data-driven target selection approach provides new insights for PET imaging of pancreatic cancer and lays the foundation for future development of PET imaging probes targeting different molecules [120].

### Multimodal imaging

The diagnosis of pancreatic cancer remains challenging due to the inherent limitations of individual imaging modalities, which can hinder early detection and precise disease characterization [46]. To address these challenges, multimodal imaging has emerged as a powerful approach, integrating two or more imaging techniques to capitalize on their respective strengths while mitigating their limitations. This synergistic strategy enables a more comprehensive assessment of tumor biology and pathology, thereby enhancing diagnostic accuracy and improving clinical decision-making [121]. A major advancement in multimodal imaging is the development of multifunctional nanomaterials capable of integrating multiple imaging modalities within a single platform. These nanostructures are engineered to enhance imaging precision, providing high-resolution, multimodal contrast that improves both sensitivity and specificity in pancreatic cancer detection [122]. By incorporating functionalities such as fluorescence, magnetic resonance imaging (MRI), positron emission tomography (PET) and ultrasound, these nanomaterials enable more precise tumor localization and characterization, even at early disease stages [32]. This chapter will focus on recent developments in nanomaterial-based multimodal imaging technologies, exploring their potential to enhance early diagnosis, improve tumor characterization and facilitate more personalized treatment approaches for pancreatic cancer.

**Figure 6.**
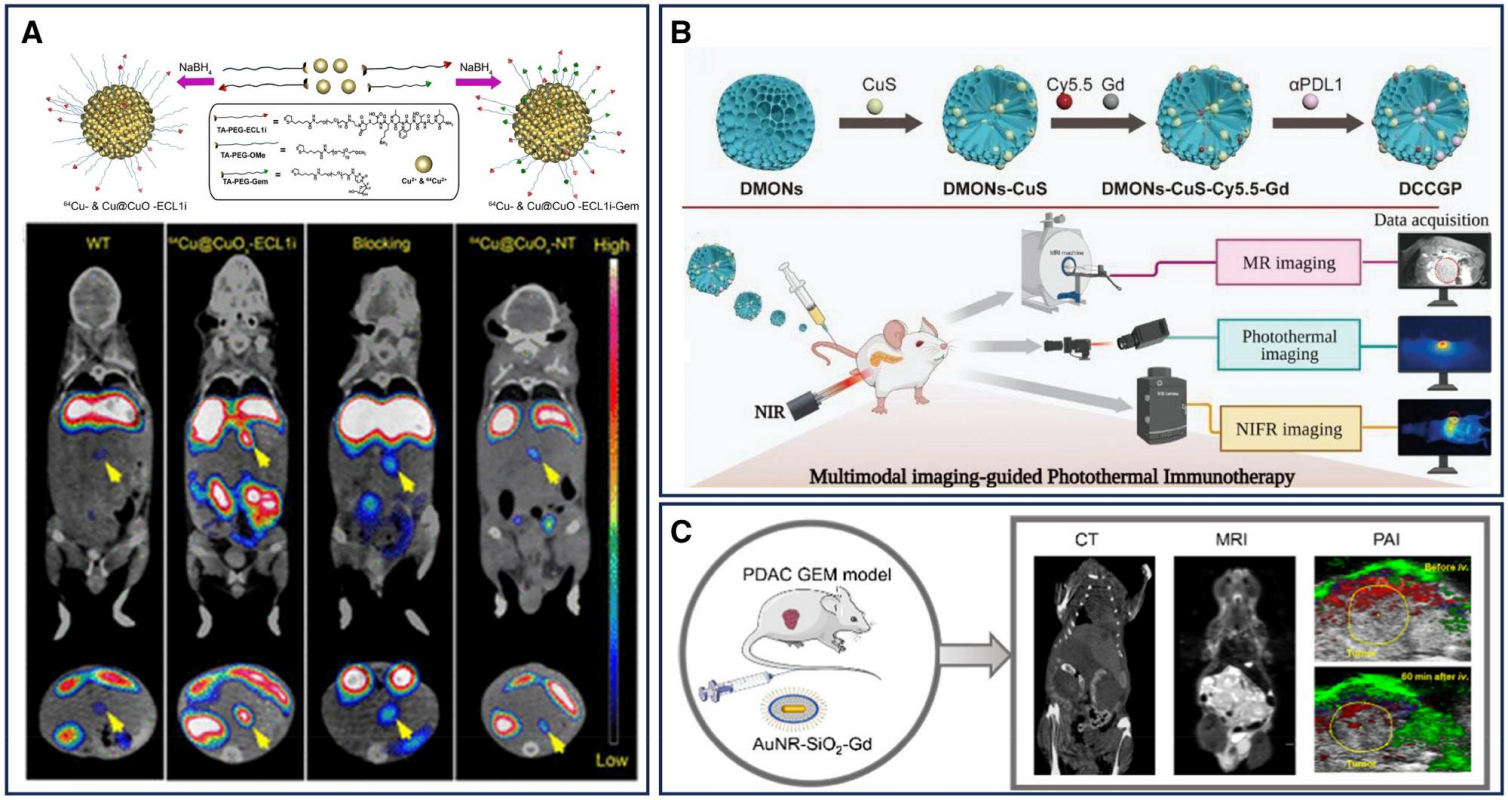
Nanomaterial-assisted multimodal imaging for the diagnosis of pancreatic cancer. (**A**) CC chemokine receptor 2-targeted copper nanoparticles for PET/CT-guided gemcitabine delivery in pancreatic ductal adenocarcinoma. Reproduced with permission [126]. Copyright 2021, American Chemical Society. (**B**) Organic silica for multimodal imaging-guided photoimmunotherapy of pancreatic cancer. Reproduced with permission [127]. Copyright 2023, Wiley‐VCH GmbH. (**C**) Multimodal imaging of pancreatic cancer using AuNR-SiO_2_-Gd multifunctional nanomaterials as contrast agents. Reproduced with permission [128]. Copyright 2020, American Chemical Society.

The development of combined positron emission tomography and computed tomography (PET-CT) by Townsend and colleagues in collaboration with Siemens Medical in 1998 marked a pivotal advancement in multimodal imaging technology [123]. By integrating the anatomical precision of CT with the molecular insights of PET, this hybrid imaging modality revolutionized oncologic diagnostics, enabling more accurate tumor detection and characterization. PET-CT has since become an indispensable tool in pancreatic cancer imaging, providing critical information for tumor staging, therapeutic response assessment and recurrence monitoring [124]. The use of radiolabeled glucose tracers, such as ^18^F-FDG, has significantly improved pancreatic cancer detection. The increased metabolic activity of pancreatic tumor cells leads to enhanced ^18^F-FDG uptake, allowing for the visualization of even small malignancies [125]. Beyond ^18^F-FDG, other radioisotopes, including ^64^Cu, have been explored to enhance PET-CT imaging sensitivity and specificity. Recently, Liu *et al*. developed an ultrasmall copper-based nanomaterial (Cu@CuOx) targeting the CCR2 receptor for PET-CT imaging of pancreatic cancer. This system leverages the intrinsic radiolabeling capabilities of ^64^Cu, enabling high-specific-activity PET imaging. The ultrasmall nanoparticles exhibit favorable biodistribution and rapid systemic clearance, reducing toxicity while enhancing tumor specificity. In KPPC and KPC mouse models, ^64^Cu-Cu@CuOx-ECL1i demonstrated selective tumor accumulation with minimal nonspecific retention. Compared to ^18^F-FDG, this nanoplatform exhibited a higher tumor-to-muscle ratio, underscoring its potential for early and sensitive detection of pancreatic ductal adenocarcinoma (Figure 6A) [126].

Despite its advantages, PET-CT imaging is limited by the relatively short tracking time imposed by radioisotope half-lives and the requirement for specialized radiochemical facilities. However, hybrid PET-CT systems incorporating radiolabeled nanoparticles offer a promising approach for quantitatively assessing nanoparticle distribution and anatomical structures within targeted tissues. This advancement holds significant potential for precision drug delivery. A recent study demonstrated the utility of superparamagnetic nanoporous silicon nanoparticles labeled with gallium-68 (^68^Ga) for PET-CT imaging of pancreatic cancer. These nanoparticles not only function as contrast agents but also serve as drug carriers for gemcitabine, effectively integrating diagnostic and therapeutic functions within a single platform. Further investigations into PEGylation and surface modifications have optimized pharmacokinetics and biodistribution, enhancing tumor-specific accumulation while minimizing off-target effects, thereby improving both imaging precision and therapeutic efficacy [129].

In recent years, magneto-optical nanoplatforms integrating near-infrared fluorescence, photoacoustic imaging and magnetic resonance imaging have attracted significant attention for their potential in medical diagnostics [130, 131]. These multimodal imaging systems offer distinct advantages over conventional nanomaterials by combining complementary imaging techniques, thereby enhancing spatial resolution and diagnostic accuracy. Magneto-optical nanomaterials are typically engineered with fluorescent dyes and MRI contrast agents, leveraging both magnetic and optical properties to facilitate precise disease targeting. Through magnetic guidance and specific ligand interactions, these platforms enable both diagnostic imaging and targeted drug delivery. Their ability to integrate multiple imaging modalities positions magneto-optical nanoprobes as promising candidates for the early detection and treatment of pancreatic cancer [132, 133].

Ling *et al*. developed modular, self-assembled dendritic molecular nanomaterials (Gd-1@ and DiR/Gd-1@) for dual-modality imaging of pancreatic cancer using MRI and near-infrared fluorescence. These nanomaterials exhibited high MRI relaxivity (*r*_1_ = 5.9 mM^−1^s^−1^), nearly twice that of the clinically approved contrast agent Gd-DOTA. The encapsulation of the near-infrared fluorescence dye DiR within the nano-micelles formed by Gd-1 provided spatial isolation from the MRI contrast agent, reducing mutual interference and enhancing the efficiency of dual-modality imaging [50].

Further advancements have been achieved with organosilicon nanoparticles, which offer multifunctional diagnostic and therapeutic capabilities. Wang *et al*. developed a multimodal imaging-guided organosilicon nanomedicine (DCCGP) for photoimmunotherapy in pancreatic cancer. This nanoplatform integrates fluorescence, MRI and real-time infrared photothermography, significantly improving both diagnostic precision and therapeutic efficacy. The incorporation of copper sulfide nanoparticles enhanced tumor permeability and enabled external modulation of immunotherapy. DCCGP demonstrated a high MRI relaxivity (*r*_1_ = 6.426 mM^−1^s^−1^) and exhibited a concentration-dependent T_1_ signal intensity, underscoring its potential as a highly effective imaging and therapeutic tool for pancreatic cancer (Figure 6B) [127].

In addition to magneto-optical nanoplatforms, a diverse range of multimodal imaging strategies has been developed to enhance the diagnosis and treatment of pancreatic cancer. By integrating multiple imaging modalities within a single system, these approaches leverage the unique advantages of each technique to improve tumor detection, characterization and therapeutic monitoring. Zhao *et al*. developed a core-shell gold nanorod-silica-gadolinium (AuNR-SiO_2_-Gd) nanomaterial that functions as a contrast agent for enhanced MRI, CT and PA imaging. Although the abundant tumor stroma and sparse vascular distribution in pancreatic cancer limit the distribution of the nanomaterial within the tumor, its high accumulation in the surrounding soft tissues and sparse distribution within the tumor create a negative contrast in CT/PAI and a positive contrast in MRI. This difference in contrast helps to achieve clear imaging of the tumor within the complex pancreatic tumor microenvironment. This multimodal nanoplatform enables a more comprehensive and precise assessment of tumor morphology and vascularization, thereby improving diagnostic accuracy (Figure 6C) [128]. Similarly, Hassan and colleagues designed a multifunctional nanoplatform based on hollow mesoporous silica nanoparticles, termed Gem-PFH-Au star-HMS-IGF1. This system integrates US, CT and PA imaging for high-resolution tumor visualization while simultaneously enabling photothermal therapy for targeted tumor ablation in a patient-derived xenograft model of pancreatic cancer. By combining diagnostic and therapeutic functionalities, these advanced nanoplatforms represent a promising direction for precision oncology [110].

Despite significant progress in multimodal imaging for pancreatic cancer diagnosis, several technical challenges persist. These include the need for faster imaging acquisition, improved spatial resolution, enhanced signal-to-noise ratio and greater device portability. Addressing these limitations is critical for advancing the clinical utility of multimodal imaging in pancreatic cancer detection and management. Nanomaterial-based imaging platforms offer promising solutions by enabling the integration of multiple contrast agents within a single system. This approach facilitates tumor-specific imaging, allowing for early detection, precise monitoring of metastatic progression and improved therapeutic planning. The continued development of these advanced imaging technologies holds great potential for transforming the clinical management of pancreatic cancer, ultimately improving patient outcomes in this highly aggressive malignancy.

### Liquid biopsy

Although nanomedicine-guided imaging technologies have played important roles in tumor localization, staging and assessment of resectability, they are often invasive, which may cause patient discomfort and cannot directly reflect the molecular characteristics of tumors. In recent years, liquid biopsy has gradually attracted attention due to its minimally invasive nature and the ability to continuously monitor cancer progression. Liquid biopsy acquires information on tumor genomics, transcriptomics and proteomics by detecting biomarkers such as circulating tumor cells (CTCs), circulating tumor DNA (ctDNA), noncoding RNAs (ncRNAs) and extracellular vesicles (EVs) in blood or other body fluids. This provides new possibilities for the early diagnosis of pancreatic cancer, therapeutic strategies, drug resistance, recurrence monitoring and prognostic assessment [134]. The introduction of nanomaterials in liquid biopsy has demonstrated unique advantages, mainly reflected in high sensitivity and specificity, multiplex biomarker detection and innovative detection methods. For example, nanomaterial-based sensors can detect low-abundance ctDNA and minute miRNAs in the blood, and simultaneously detect multiple biomarkers, thereby more comprehensively reflecting the molecular characteristics of tumors [135]. Nanopatterned biochips can detect a variety of noncoding RNAs (ncRNAs), which helps to gain a more comprehensive understanding of the heterogeneity and dynamic changes of tumors [136]. EVs are nanoscale vesicles released by tumor cells and carry a wealth of biological information, such as proteins, RNA and DNA fragments. They are important biomarkers in liquid biopsies. Surface-enhanced Raman spectroscopy (SERS) based on nanomaterials can achieve highly sensitive detection of EVs. Through SERS technology, biological molecules within EVs can be more efficiently detected and analyzed, providing a new means for the early diagnosis of pancreatic cancer [137]. To achieve highly sensitive detection of the pancreatic cancer biomarker CA19-9, Kim *et al*. developed a novel SERS-based liquid biopsy method. By embedding silver nanogap shells (AgNSM) into mesoporous silica nanoparticles, they successfully synthesized SERS-active nanoprobes that emit stable and intense SERS signals. These nanoprobes not only exhibit strong SERS signals but also demonstrate excellent long-term stability, maintaining signal stability without the need for an additional protective layer. When conjugated with antibodies, these nanoprobes were applied in SERS immunoassays and successfully detected CA19-9 concentrations as low as 0.025 U/mL, which is significantly lower than the detection limit of traditional enzyme-linked immunosorbent assay (ELISA) (0.3 U/mL). The concentration of CA19-9 was successfully quantified through changes in SERS signal intensity, providing important information for the diagnosis of pancreatic cancer [138]. In addition, to further improve detection efficiency and reduce sample volume and detection time, researchers have employed fluorescently encoded quantum dots and rare-earth-doped nanoparticles to achieve multiplex detection from a single-tube sample. These nanoparticles can specifically bind to ctDNA, miRNA and circulating proteins (such as PD-L1). Through spectral unmixing techniques, multiple biomarkers can be detected simultaneously, thereby obtaining information on various indicators from a single sample tube [134]. It is undeniable that nanomaterials, through their extreme sensitivity design and spatiotemporal multi-omics capabilities, are redefining the boundaries of liquid biopsy. Their core advantages lie not only in performance enhancement but also in the creation of entirely new detection dimensions that traditional technologies cannot reach (such as real-time *in vivo* monitoring and single-cell multi-omics). These advancements provide the foundation for pancreatic cancer diagnosis to transition from static snapshot analysis to dynamic panoramic insight.

## Nanomaterial-assisted precision therapy of pancreatic cancer

The dense stromal architecture, poor vascularization and high metabolic activity of pancreatic cancer present significant barriers to effective drug delivery, often limiting the efficacy of conventional therapeutic strategies. These challenges contribute to poor drug accumulation within the tumor microenvironment, leading to unsatisfactory treatment outcomes [6]. Nanomaterials offer a promising solution by overcoming these physiological barriers and enhancing drug delivery efficiency. These innovative nanoplatforms covalently link or physically encapsulate therapeutic components (such as chemotherapeutic drugs, gene-editing tools or bioactive molecules) within biocompatible nanocarriers. This approach not only achieves stable delivery of the payload but also significantly enhances its resistance to enzymatic degradation/biodegradation. Moreover, surface functionalization strategies (such as PEGylation or grafting of targeting ligands) effectively inhibit nonspecific adsorption and off-target effects, thereby greatly improving the targeting accumulation and bioavailability of therapeutic agents at the lesion site [139]. This is attributed to the nanoscale size effect (1–100 nm). Such intelligent delivery systems can not only cross multiple biological barriers and interact specifically with subcellular organelles (such as lysosomes and mitochondria) but also activate key intracellular signaling pathways (such as apoptosis pathways and autophagy regulation) through spatiotemporally controlled drug release mechanisms, thereby achieving precise intervention in the life cycle of tumor cells [140]. Additionally, nanoplatforms enable the simultaneous delivery of multiple therapeutic agents, including chemotherapy, immunotherapy and gene therapy, thereby promoting a multimodal and synergistic treatment approach. Beyond their role in drug delivery, nanomaterials can also function as theranostic agents, integrating both diagnostic and therapeutic capabilities to enable real-time monitoring and precision treatment of pancreatic cancer [141]. This section will focus on the recent research progress of nanomaterial-based strategies designed to overcome tumor-associated barriers and enhance targeted drug delivery for more effective precision therapy in pancreatic cancer.

### Chemodynamic therapy

Chemodynamic therapy (CDT) is an emerging cancer treatment strategy that exploits endogenous hydrogen peroxide (H_2_O_2_) and other tumor microenvironmental substrates to generate cytotoxic hydroxyl radicals (·OH) through Fenton and Fenton-like reactions. This localized production of reactive oxygen species selectively induces tumor cell death while minimizing adverse effects on normal tissues, offering a highly specific and promising approach for cancer therapy [142–144]. The intrinsic selectivity and reduced systemic toxicity of CDT highlight its potential for clinical translation, positioning it as a novel strategy for precision oncology [145]. However, the TME of pancreatic cancer is characterized by low pH, low H_2_O_2_ content and high expression of reducing substances, all of which pose significant challenges to the efficacy of CDT. Therefore, a series of nanomaterials have been developed as nanoplatforms to overcome the challenges brought by the TME to CDT. First, nanomaterials can regulate the local pH value to make it closer to the optimal conditions for the Fenton reaction, and at the same time, pH-responsive materials are designed to efficiently release Fenton catalysts (such as Fe^2+^ and Cu^+^) in the weakly acidic TME. Second, some nanomaterials can act as self-suppliers of H_2_O_2_, or increase the H_2_O_2_ content in tumor cells by combining with other therapeutic methods, providing sufficient substrates for the Fenton reaction. In addition, nanomaterials can also consume reducing substances in tumor cells, reducing their consumption of hydroxyl radicals (•OH), thereby enhancing the oxidative damage effect of CDT [146]. These mechanisms work together to enable nanomaterials to show great therapeutic potential in CDT for pancreatic cancer, laying the foundation for clinical applications.

**Figure 7.**
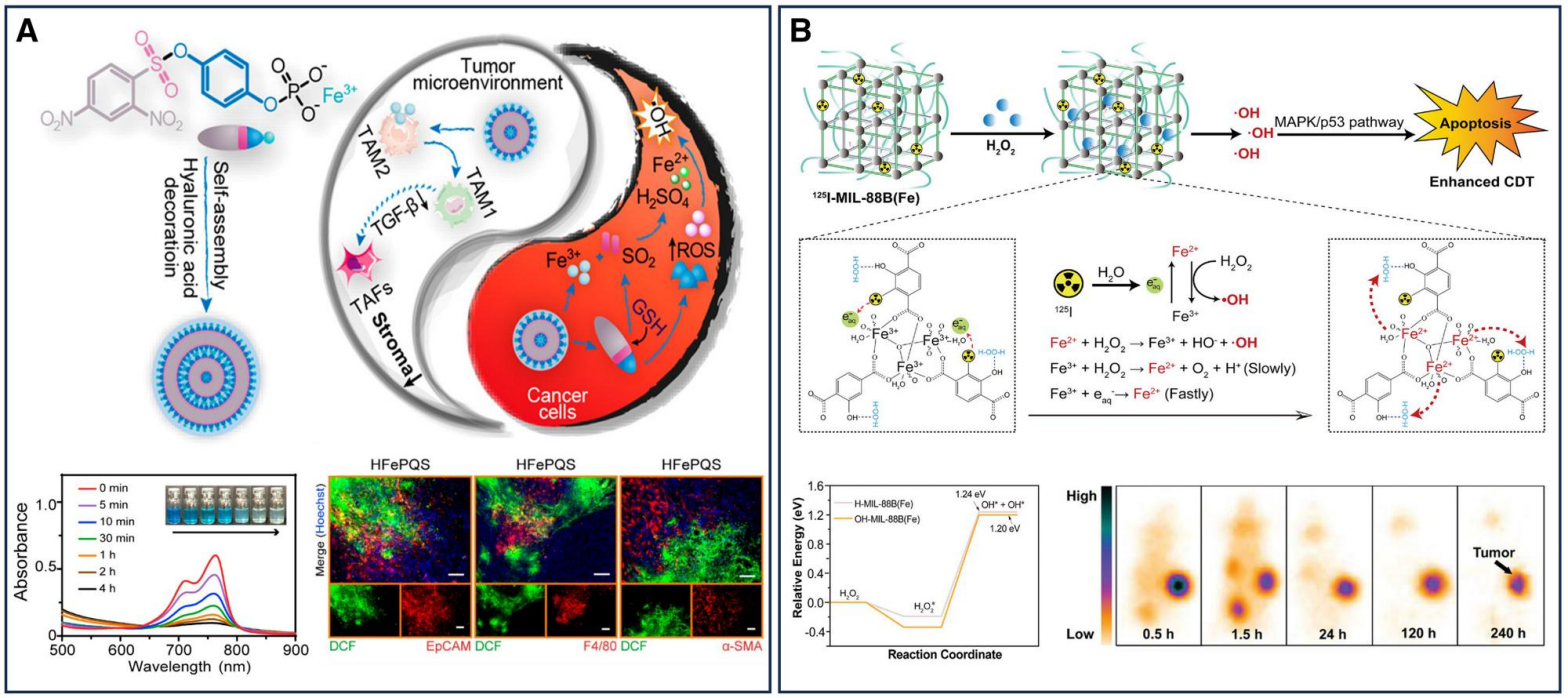
Nanomaterial-assisted chemodynamic therapy of pancreatic cancer. (**A**) HFePQS as a chemodynamic nanocomplex used to improve pancreatic cancer therapy. Reproduced with permission [148]. Copyright 2020, American Chemical Society. (**B**) Radioactive iodine promoting transition metal valence changes for enhanced chemodynamic treatment of pancreatic cancer. Reproduced with permission [149]. Copyright 2024, Wiley-VCH GmbH.

The glutathione concentrations in pancreatic cancer cells are approximately 5–10 times higher than those found in normal cells. This elevated GSH level within the tumor microenvironment effectively scavenges·OH and mitigates oxidative stress, thereby creating a significant barrier to the efficacy of CDT [147]. To overcome this challenge, Chen *et al*. developed a nanocomplex, HFePQS, synthesized via the self-assembly of 4-(phosphonooxy) phenyl-2,4-dinitrobenzenesulfonate and Fe³^+^, followed by surface functionalization with hyaluronic acid for targeted CDT in pancreatic cancer. This nanoplatform employs a GSH-sensitive mechanism to regulate the controlled release of therapeutic agents while selectively activating the Fenton reaction. The colorimetric shift observed in methylene blue (MB) assays confirms the ability of HFePQS to initiate the Fenton reaction, inducing oxidative stress and apoptosis in cancer cells. Additionally, this strategy facilitates tumor-associated macrophage reprogramming and stromal cell inactivation, further disrupting the tumor microenvironment. Compared to conventional gemcitabine-based chemotherapy, this CDT nanoplatform demonstrates superior therapeutic efficacy against pancreatic cancer, highlighting its potential as an innovative treatment strategy (Figure 7A) [148].

Despite its therapeutic potential, CDT faces several challenges in pancreatic cancer treatment. One of the primary limitations is the insufficient endogenous hydrogen peroxide available to sustain hydroxyl radical production within the tumor microenvironment, resulting in suboptimal cytotoxic effects [150]. To address this limitation, various strategies have been explored to elevate H_2_O_2_ levels in pancreatic cancer cells and enhance CDT efficacy. One such approach involves the use of DNAzyme to selectively cleave catalase (CAT)-related RNA, thereby downregulating CAT activity and increasing intracellular H_2_O_2_ concentrations [151]. Furthermore, copper has demonstrated superior catalytic efficiency in Fenton-like reactions compared to other transition metals, making it an attractive candidate for CDT enhancement. Leveraging these properties, researchers have developed a Cu and DNAzyme-mediated nanomaterial to synergistically enhance CDT through the integration of cuproptosis copper-induced form of regulated cell death. Fluorescence probe analysis and nanovector-based studies have confirmed that this nanoplatform effectively upregulates intracellular H_2_O_2_ levels. By facilitating the cyclic redox transformation between Cu and Cu^2+^ via glutathione and Fenton-like reactions, this strategy amplifies oxidative stress, achieving a potent therapeutic effect in pancreatic cancer. The integration of CDT with copper-mediated oxidative mechanisms offers a promising avenue for improving pancreatic cancer treatment [152].

The conventional methods employed to enhance CDT typically involve the utilization of reducing agents, such as GSH and tannins, which induce valence changes in transition metals. However, the inherent instability, unsustainable electron supply and challenges in precisely controlling these substances have limited their clinical applicability [153]. To overcome these limitations, researchers are exploring more stable electron sources, including radionuclides, as a means of optimizing CDT efficacy. Radioactive iodine-125 (^125^I), a medical radionuclide with a half-life of 60 days, has been identified as a promising candidate due to its ability to continuously generate hydrated electrons. This sustained electron supply facilitates transition metal valence cycling, thereby enhancing CDT efficiency. In a recent study, Bu *et al*. leveraged this property by developing iron-based metal-organic framework (MOF) nanoparticles labeled with ^125^I to augment CDT in pancreatic cancer. The porous architecture of these nanoparticles promotes H_2_O_2_ accumulation, thereby amplifying the Fenton reaction. Furthermore, the continuous generation of hydrated electrons by ^125^I enhances Fe³^+^ to Fe^2+^ conversion, further promoting ·OH production and intensifying oxidative stress within the tumor microenvironment (Figure 7B). This dual enhancement mechanism has demonstrated significant antitumor efficacy in preclinical pancreatic cancer models, highlighting the potential of radionuclide-assisted CDT as a novel therapeutic strategy [149].

### Light-based therapy

Despite significant advancements in CDT, its therapeutic efficacy remains limited, and resistance mechanisms continue to pose challenges in pancreatic cancer treatment. To overcome these limitations, phototherapy has emerged as a promising alternative, offering high selectivity, minimal invasiveness and real-time treatment monitoring [154]. Light-based therapeutic modalities, including photothermal therapy (PTT) and photodynamic therapy (PDT), utilize specific wavelengths of light in combination with photosensitizers to selectively target and destroy cancer cells. These approaches induce cytotoxic effects through distinct mechanisms: PTT generates localized hyperthermia, while PDT produces ROS in the presence of oxygen. The ability to precisely control these therapeutic effects through external light activation makes phototherapy a highly attractive strategy for pancreatic cancer treatment [155–157].

PTT is a highly promising novel approach for tumor treatment, leveraging nanomaterials with high photothermal conversion efficiency to selectively induce tumor cell death. The core mechanism involves the accumulation of photothermal agents within tumor tissues, followed by irradiation with near-infrared light. These nanomaterials absorb NIR radiation and convert it into heat energy, rapidly elevating the local temperature within the tumor microenvironment [158–160]. This thermal effect triggers a heat shock response in tumor cells, leading to apoptosis or necrosis. Additionally, PTT-induced hyperthermia can disrupt tumor vasculature, impairing nutrient supply and further contributing to tumor regression [161]. A range of nanomaterials, including gold nanoparticles, carbon nanotubes and graphene, have been extensively investigated as photothermal conversion agents due to their ability to efficiently absorb NIR light and generate localized hyperthermia exceeding 42°C. PTT has demonstrated considerable potential for tumor ablation, with the capacity to induce substantial tumor reduction or even complete eradication [162].

However, the application of PTT in pancreatic cancer presents significant challenges due to the aggressive nature of the disease and its intrinsic resistance to therapeutic interventions. The pancreatic tumor microenvironment is characterized by extensive fibrosis, limited immune infiltration and severe hypoxia, all of which may compromise the efficacy of PTT. To address these limitations, researchers are exploring the integration of nanomaterials in PTT to enhance therapeutic outcomes, improve tumor penetration and overcome microenvironmental barriers, offering new possibilities for the treatment of pancreatic cancer [163].

**Figure 8.**
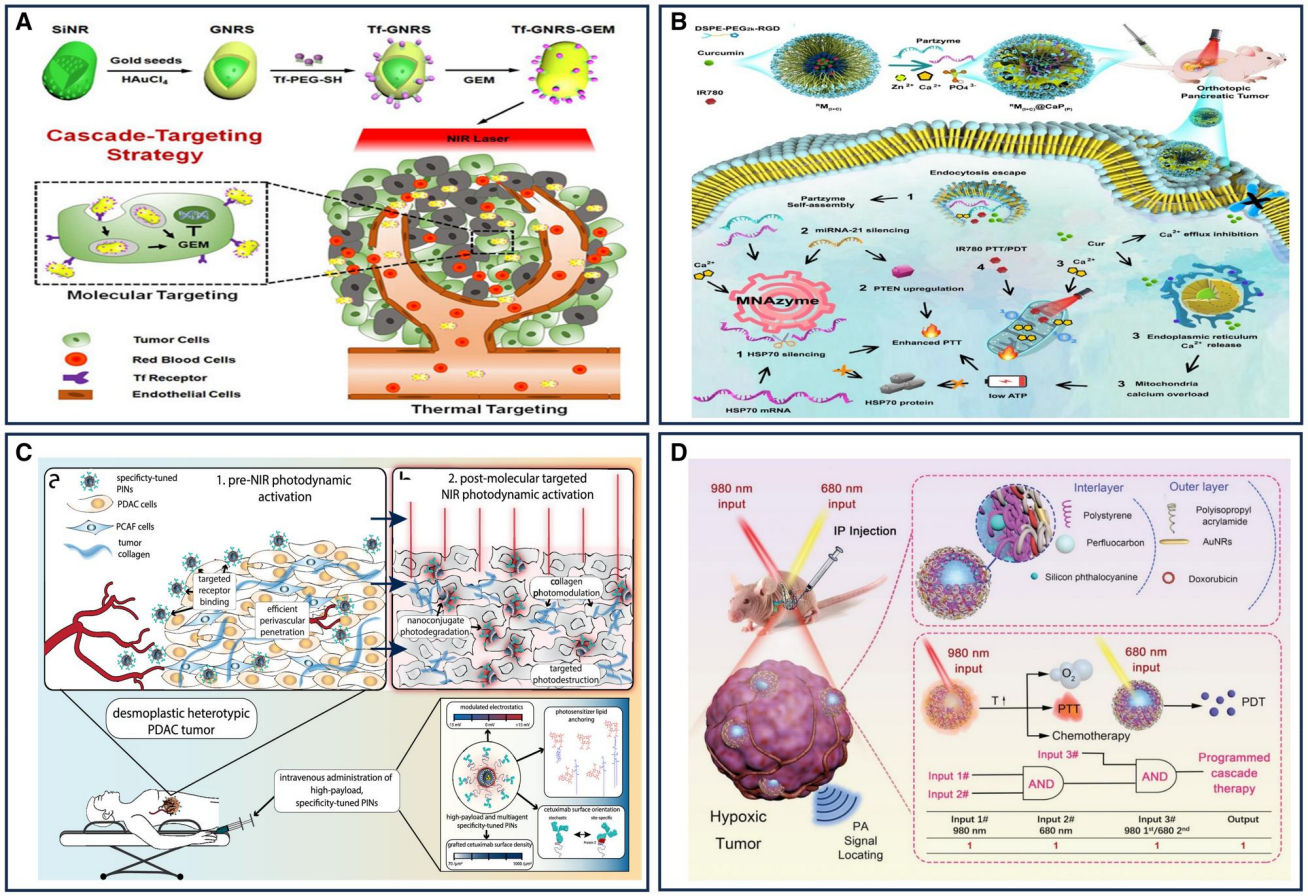
Nanomaterial-assisted light-based therapy of pancreatic cancer. (**A**) GNRS nanomaterial for cascade tumor targeting and enhanced photothermal-gemcitabine chemotherapeutic effects under NIR. Reproduced with permission [164]. Copyright 2017, American Chemical Society. (**B**) Multicomponent DNAzyme nanomachine for tumor-specific photothermal therapy of pancreatic cancer and triggering ROS-mediated photodynamic therapy. Reproduced with Permission [165]. Copyright 2023, Springer Nature. (**C**) Specifically tuned PIN is activated by molecularly targeted NIR photodynamic activation to achieve precise photodestruction of PDAC, a heterogeneous desmoplastic cell of pancreatic cancer tissue. Reproduced with permission [166]. Copyright 2019, American Chemical Society. (**D**) PSPP-Au_980_-D effective inhibition of hypoxic pancreatic tumors by programmed Cascade therapy via NIR-II irradiation. Reproduced with permission [167]. Copyright 2021, Wiley-VCH GmbH.

To overcome the tumor microenvironmental barriers associated with pancreatic cancer, Nie *et al*. developed a composite nanoplatform comprising a gold nanoshell-encapsulated rod-shaped mesoporous silica nanoparticle (Tf-GNRS) loaded with gemcitabine (Tf-GNRS-GEM). This multifunctional system leverages the tumor-specific photothermal effect to enhance local blood perfusion and increase vascular permeability, thereby promoting nanoparticle accumulation and penetration within the tumor microenvironment. By disrupting the dense stromal barrier characteristic of pancreatic cancer, this nanoplatform facilitates deeper tumor infiltration, improving photothermal targeting efficiency. The enhanced nanoparticle accumulation leads to a higher concentration of Tf-GNRS within the tumor, significantly augmenting the therapeutic efficacy of pancreatic cancer treatment (Figure 8A) [164]. In cancer therapy, tumor cells activate intrinsic self-defense mechanisms, such as the upregulation of heat shock proteins, in response to therapeutic stress. This adaptive response can significantly reduce treatment efficacy and contribute to tumor recurrence and metastasis [168]. The ability of tumors to evade therapy by inducing such mechanisms presents a significant challenge in the effective treatment of pancreatic cancer. By leveraging the tumor’s unique microenvironmental properties, such as altered blood flow and increased permeability, this composite nanoplatform not only improves drug delivery but also potentiates the therapeutic effects, paving the way for more effective treatments of pancreatic cancer in the future.

To mitigate the inhibitory effects of HSPs on photothermal therapy (PTT), Zhang *et al*. developed a multicomponent deoxyribozyme (MNAzyme)-powered nanomachine designed to cleave HSP70 mRNA prior to PTT. This approach effectively disrupts the protective role of HSPs, enhancing the therapeutic impact of PTT in pancreatic cancer. The nanomachine was synthesized by mineralizing MNAzyme onto the polyethylene glycol (PEG) layer of distearoylphosphatidylethanolamine (DSPE)-PEG micelles, which were modified with arginine-glycine-aspartic acid (RGD) for tumor targeting. These micelles were co-loaded with the PTT dye IR780 and the calcium efflux pump inhibitor curcumin. Under acidic tumor microenvironment conditions, the nanomachines release MNAzyme and the cofactor Ca^2+^, triggering self-assembly into an active form that utilizes oncogenic microRNA-21 (miRNA-21) to cleave HSP mRNA. Additionally, silencing miRNA-21 upregulates the tumor suppressor gene PTEN, further sensitizing tumor cells to PTT. Curcumin enhances this effect by maintaining elevated intracellular Ca^2+^ levels and inhibiting the heat chaperone protein ATP through disruption of mitochondrial Ca^2+^ homeostasis. This multitargeted approach enhances IR780-mediated PTT through a threefold mechanism: suppression of HSP70, upregulation of PTEN and mitochondrial Ca^2+^ dysregulation. Both *in vitro* and *in vivo* studies demonstrated that MNAzyme-based nanomachines effectively modulate HSP and PTEN expression, leading to significant tumor suppression following laser irradiation (Figure 8B) [165].

In contrast to photothermal therapy, which relies on localized hyperthermia, photodynamic therapy exerts its cytotoxic effects through the generation of reactive oxygen species [154]. The first clinical trial of PDT for pancreatic cancer was conducted by Bown *et al*. in 2002, marking a milestone in the exploration of light-based therapeutic strategies [169]. Since then, hundreds of photosensitizers have been investigated in clinical and preclinical studies, broad diverse molecular classes such as porphyrins, chlorins and phthalocyanine derivatives. Upon light activation, photosensitizers absorb energy, resulting in the excitation of electrons within their molecular structure. The energy transfer to molecular oxygen generates highly reactive oxygen radicals, which induce oxidative damage and ultimately trigger apoptosis in cancer cells [170]. Despite its therapeutic potential, the clinical translation of PDT for pancreatic cancer remains challenging. Major limitations include suboptimal photosensitizer delivery, insufficient tumor oxygenation and intrinsic resistance mechanisms in aggressive tumor phenotypes. Addressing these challenges requires innovative strategies to enhance photosensitizer accumulation, improve oxygen availability and overcome tumor adaptive resistance, thereby optimizing PDT efficacy in pancreatic cancer treatment. The introduction of nanomaterials provides new ideas for solving these problems. By encapsulating PS, nanomaterials can significantly enhance their solubility stability and inhibit self-quenching, thereby improving the generation efficiency of ^1^O_2_. In addition, nanomaterials can also be loaded with oxygen carriers or *in situ* oxygen-generating systems to effectively alleviate the hypoxic state of tumors, thereby synergistically optimizing the efficacy of PDT [171].

As clinical PDT protocols continue to evolve, increasing PS dosages and expanded irradiation areas have raised concerns regarding potential adverse effects, including off-target phototoxicity. In pancreatic cancer, the risk of collateral damage, such as gastrointestinal hemorrhage, is particularly concerning when illumination extends into critical anatomical regions. To overcome these challenges, researchers have developed a range of nanomaterial-based strategies to optimize photosensitizer delivery, minimize accumulation in healthy tissues and reduce systemic photosensitivity, thereby improving treatment safety and efficacy [172, 173]. Tayyaba Hasan designed a photodynamic immunotherapy nanoconjugate (PIN) system for pancreatic cancer treatment, utilizing cetuximab (Cet), an anti-epidermal growth factor receptor (EGFR) monoclonal antibody, to enhance tumor targeting. The PINs were systematically optimized through multivariate adjustments, including PS lipid anchoring, electrostatic modulation, Cet orientation and surface density, to improve tumor penetration and binding specificity. In a stroma-rich pancreatic cancer model, these nanoconjugates exhibited deep tumor infiltration, inducing extensive tumor necrosis upon photodynamic activation. The Cet-PINs demonstrated a 16.9-fold increase in binding specificity and enhanced molecularly targeted near-infrared photodynamic cytotoxicity by 16-fold. Furthermore, they achieved extravascular penetration up to 470 micrometers and significantly reduced collagen density within the tumor microenvironment, thereby potentially mitigating the fibrotic response that often limits drug diffusion. This approach not only improved the therapeutic precision of PDT but also enhanced its safety profile, underscoring the potential of nanomaterial-engineered photodynamic immunotherapy for pancreatic cancer treatment (Figure 8C) [166].

The efficacy of photodynamic therapy (PDT) relies on the generation of singlet oxygen (^1^O_2_), a process that is inherently oxygen-dependent. However, the hypoxic nature of the pancreatic tumor microenvironment poses a significant challenge, often limiting the therapeutic effectiveness of PDT. To address this limitation, oxygen-carrying nanomaterials, particularly perfluorocarbons (PFCs), have gained increasing attention for their potential to enhance PDT outcomes. PFC-based nanomaterials offer several advantages, including high oxygen solubility, substantial porosity and favorable biocompatibility, making them promising candidates for improving oxygen availability in hypoxic tumors [172]. Li *et al*. developed a functionalized multilayer polymer nanoplatform, PFC/SiPc@PS@PNIPAM-Au980-DOX (PSPP-Au980-D), designed as a near-infrared -triggered “oxygen bomb” for pancreatic cancer therapy. This system integrates AuNRs as a NIR-II photothermal agent, which, upon 980 nm laser irradiation, generates localized heat to induce oxygen release from the PFC core. This process alleviates tumor hypoxia while simultaneously triggering localized hyperemia and controlled release of doxorubicin. A subsequent 680 nm laser irradiation activates the photosensitizer, facilitating singlet oxygen generation and enabling photodynamic radiotherapy within the oxygen-enriched tumor microenvironment. The photothermal conversion efficiency of PSPP-Au980 reaches 40.62%, with a temperature increase of 14.1°C observed at a tissue depth of 4 mm under 980 nm laser exposure. *In vivo* studies demonstrated that the programmed cascade therapy (980 nm/680 nm) resulted in near-complete tumor eradication within 14 days, achieving a therapeutic efficacy five times greater than single-laser irradiation strategies. This sequential treatment approach represents an innovative strategy for suppressing pancreatic tumor progression by effectively controlling oxygen release and optimizing photodynamic therapy effects (Figure 8D) [167].

### Sonodynamic therapy

Sonodynamic therapy (SDT) is a minimally invasive treatment that combines ultrasound with pharmacological agents to induce targeted tumor cell destruction. This approach involves the administration of an acoustic sensitizer, a relatively nontoxic compound that enhances cellular susceptibility to ultrasound exposure. Upon insonation, the sensitizer undergoes a transition from its ground state to an excited state, subsequently returning to the ground state and releasing energy in the process [174]. This energy transfer generates reactive oxygen species (ROS), which induce oxidative stress-mediated cell death [175]. For pancreatic cancer, is difficult to treat with conventional surgery and chemotherapy, SDT offers a potential treatment strategy. However, the therapy faces some problems, such as insufficient tumor targeting of acoustic sensitizers, and the short half-life and limited dispersal distance of reactive oxygen species, which can also limit the generation of immunogenic cell death [176].

**Figure 9.**
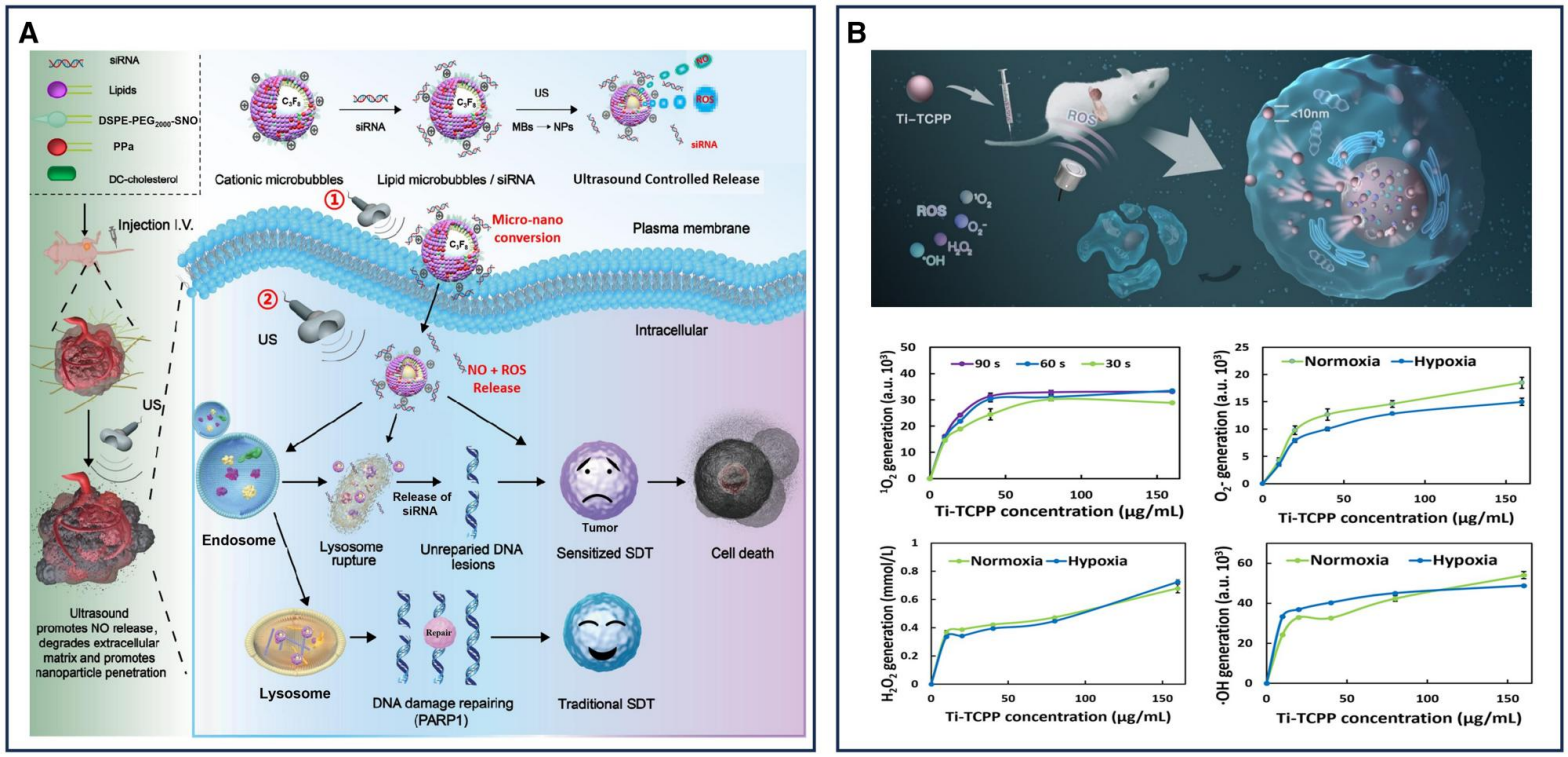
Nanomaterial-assisted sonodynamic therapy of pancreatic cancer. (**A**) Preparation of ultrasound-responsive functional microbubbles with membrane shells and adsorption of PARP1 siRNA on the surface for effective SDT of pancreatic cancer. Reproduced with permission [178]. Copyright 2024, Elsevier. (**B**) Ultra-small Ti-TCPP MOF facilitates the delivery of acoustic sensitizers and increases ROS production. Reproduced with permission [179]. Copyright 2021, Springer Nature.

Microbubbles have been approved for utilization as contrast agents in diagnostic ultrasound imaging and have also been explored as potential drug delivery vehicles. The capacity of ultrasound contrast agent microbubbles to reflect and scatter ultrasound waves, in addition to generating transient pores through the cavitation effect, is well established in the literature. This phenomenon significantly enhances the permeability of vascular endothelial cells and tumor cells, thereby mitigating the challenges associated with inadequate drug delivery and drug resistance in the treatment of pancreatic cancer using SDT [177]. In a recent publication, Liang *et al*. introduced a microbubble system referred to as PARP1 siRNA-Pyrophosphorus/SNO (P-PPaS mb). Under local ultrasound irradiation, the ROS generated by PPa not only exert tumor-killing effects in SDT but also trigger the cleavage of the SNO bond to release NO, thereby enhancing the penetration of the sonosensitizer and siRNA at the tumor sites. NO improves drug permeability by modulating the activity of matrix metalloproteinases (MMP-1 and -2), inhibiting the expression of TGF-β1, and regulating profibrotic signal transduction. Additionally, PARP1 siRNA further enhances the tumor-killing efficiency of SDT by inhibiting the DNA repair pathway. Therefore, P-PPaS MBs combined with ultrasound irradiation achieve deep pancreatic cancer therapy by enhancing drug penetration, inhibiting DNA repair, and generating highly reactive peroxynitrite (Figure 9A). In the subcutaneous SW1990 tumor model using BALB/c nude mice, the nanomaterial exhibited an inhibitory effect of up to 68.3% [178].

Although SDT has shown great potential as an emerging cancer treatment, most existing SDT methods primarily rely on the oxygen-dependent Type II SDT mode, which significantly limits its application in pancreatic solid tumors. Since Type I SDT mainly depends on the generation of cytotoxic free radicals and superoxide anions, it can kill tumors more effectively under hypoxic conditions compared to Type II SDT. Huang *et al*. developed a highly efficient mediator for type II SDT by synthesizing a metal-organic framework (MOF) platform that incorporates ultrasmall titanium-tetrakis(4-carboxyphenyl)porphyrin (TCPP) molecules, which specifically target the cell nucleus. The compact dimensions and the capacity to modulate its charge of this innovative MOF nanomaterial facilitate cellular entry, resulting in substantial production of reactive oxygen species upon exposure to low-intensity ultrasound. The Ti-TCPP MOF was observed to induce apoptosis *in vitro* by directly disrupting DNA and causing S-phase cell cycle arrest following ultrasound irradiation. Moreover, this MOF significantly inhibited *in situ* pancreatic tumor growth and extended the survival of hormonal mice following treatment with Ti-TCPP in conjunction with ultrasound (Figure 9B) [179].

### Gene therapy

In recent years, with the deepening of research in genomics and epigenomics, the treatment strategies for pancreatic cancer are undergoing transformation. Gene therapy is a medical intervention that seeks to treat diseases through the modification of a patient’s cells and DNA [180]. Before undertaking gene therapy, it is essential to conduct a thorough investigation of any potential genetic or cellular abnormalities. The corrected gene is subsequently introduced into the patient’s cells utilizing appropriate gene delivery vectors, which may include both viral and nonviral options [181]. Once inside the cell, the therapeutic gene will initiate expression, leading to the production of the intended functional protein or RNA [182, 183]. With the advancement of gene sequencing technologies, an increasing number of pancreatic cancer patients are able to identify potential therapeutic targets through genetic testing. However, most of these targets are still in the preclinical or early clinical trial stages [184]. To further realize gene therapy for pancreatic cancer, various nanomaterials have been developed as ideal carriers for gene and drug delivery. These nanomaterials can effectively address issues such as poor RNA stability, immune activation and low tumor-targeting efficiency in gene therapy, while also providing new possibilities for the clinical application of epigenomics [185].

In pancreatic cancer, almost all pancreatic cancer cells carry mutations in the KRAS gene, with a prevalence of more than 90% for the KRAS^G12D^ mutation. This mutation is a major driver of pancreatic cancer development, progression and metastasis. Therefore, inhibition of KRAS^G12D^ gene expression has become a key challenge in gene therapy for pancreatic cancer [186]. Gene silencing technology, which suppresses the expression of oncogenic genes in tumor cells and tumor stromal cells through small-molecule RNAs (such as siRNA or miRNA), has shown great potential in the treatment of pancreatic cancer. However, most RNAs have poor stability, which often leads to problems such as low tumor-targeting efficiency and insufficient cellular uptake.

**Figure 10.**
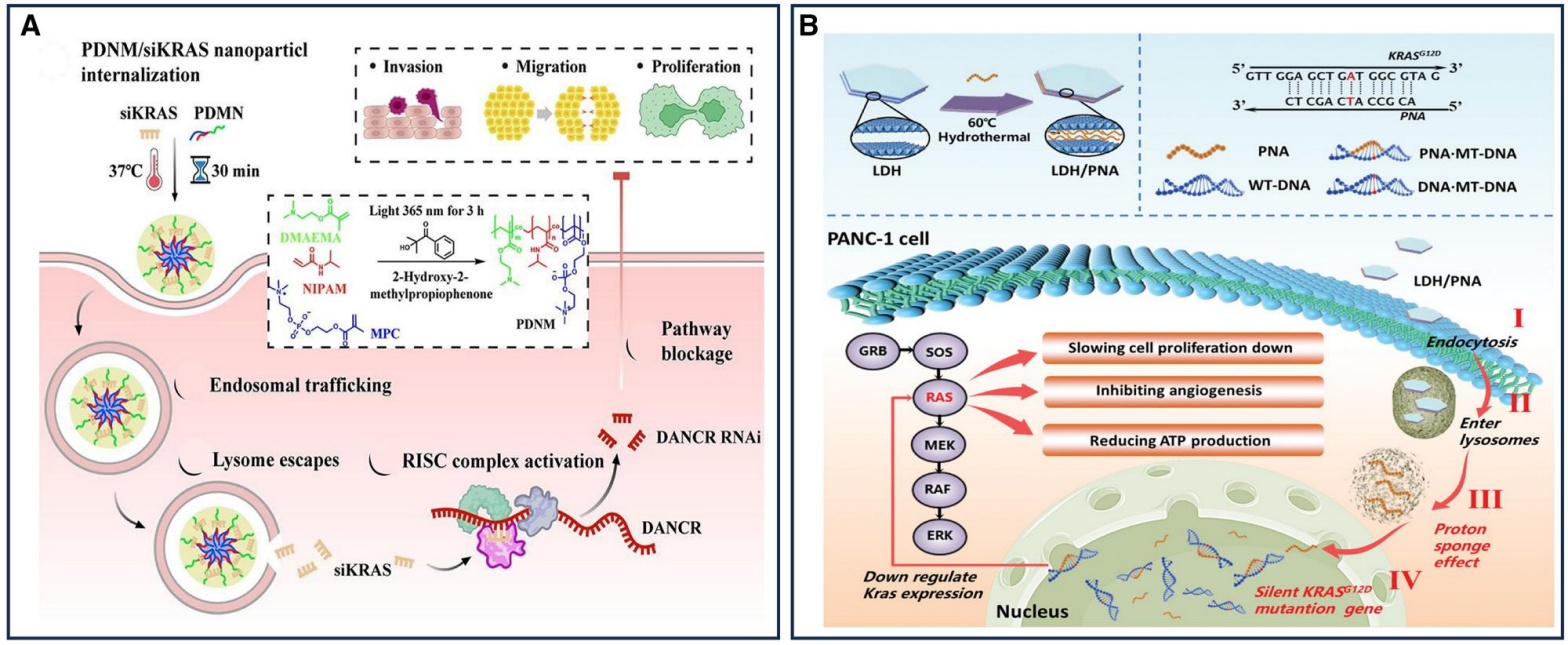
Nanomaterial-assisted gene therapy of pancreatic cancer. (**A**) PDNM as a genetic vector for key gene delivery in pancreatic cancer therapy. Reproduced with permission [188]. Copyright 2024, Elsevier (**B**) Suppression of KRAS mutation silencing by PNA enables efficient gene therapy for pancreatic cancer. Reproduced with permission [189]. Copyright 2020, Wiley-VCH verlag GmbH.

To address these issues, researchers are developing various nonviral carriers, including liposomes, polymeric nanoparticles and inorganic nanoparticles, to improve the delivery efficiency and bioavailability of RNA [187]. Tao *et al*. developed a novel triblock cationic polymer, PDMAEMA-co-PNIPAM-co-PMPC (PDNM), as an efficient gene carrier. This study achieved effective silencing of the KRAS gene via PDNM/siKRAS nanomaterials, significantly inhibiting the proliferation of pancreatic cancer cells and tumor growth. PDNM integrates the cationic properties of DMAEMA, the thermoresponsive nature of NIPAM and the cell membrane affinity of MPC, thereby significantly enhancing the cellular uptake efficiency of siRNA and enabling tumor-targeted delivery through the enhanced permeability and retention effect. The synthesis of PDNM via photoinitiated free radical polymerization is characterized by mild reaction conditions, low activation energy and rapid initiator decomposition, making it suitable for large-scale production. Experimental results demonstrated that PDNM/siKRAS nanoparticles exhibited remarkable antitumor effects in pancreatic cancer cells (AsPC-1): cell proliferation was reduced to 46.7%, Ki67 expression was decreased and apoptosis rate was increased to 44.3%. Thus, the application of PDNM as a gene carrier in pancreatic cancer therapy holds great promise (Figure 10A) [188].

Similarly inhibition of KRAS^G12D^ gene expression, Shi *et al*. proposed an efficient pancreatic cancer gene therapy based on peptide nucleic acid (PNA), utilizing a PNA-loaded layered double hydroxides (LDH) nanoplatform to target and silence the KRAS^G12D^ mutant gene. Compared with traditional DNA- or RNA-based gene therapies, PNA exhibits significant advantages in recognizing and hybridizing with target mutant sequences to form highly stable PNA-DNA hybrids. The absence of electrostatic repulsion and the constrained flexibility of the polyamide backbone notably enhance the stability of these hybrids. Moreover, the ultrasmall LDHs were specifically designed to encapsulate PNA and significantly improved the cellular uptake efficiency and nuclear enrichment of PNA through their “proton sponge effect,” thereby enabling efficient and selective targeting of intranuclear mutant sequences. Experiments demonstrated that the survival rate of mice in the LDH/PNA treatment group reached 100% over 60 days (Figure 10B) [189].

Despite some progress in gene therapy research for pancreatic cancer, the clinical significance of the majority of gene variant information remains unclear, thereby limiting the application of targeted therapy based on genetic testing in the clinical diagnosis and treatment of pancreatic cancer.

### Combined therapy

Multiple biological barriers exist in the microenvironment of pancreatic cancer, which makes it difficult to achieve effective treatment of pancreatic cancer with a single therapeutic strategy. Therefore, various comprehensive treatment strategies have emerged. These combination therapies, by integrating different therapeutic mechanisms, overcome the limitations of single-agent therapies and significantly enhance the therapeutic efficacy of pancreatic cancer [141, 171, 190]. Common combination therapies in clinical practice include chemotherapy combined with radiotherapy, chemotherapy combined with immunotherapy, and combination chemotherapy regimens with multiple drugs. For example, in locally advanced or resectable pancreatic cancer, FOLFIRINOX or gemcitabine in combination with nab-paclitaxel has been used as neoadjuvant therapy [191]. In recent years, immunotherapy has also been gradually integrated into combination treatment strategies. The combination of immune checkpoint inhibitors with chemotherapeutic agents has significantly improved the pancreatic cancer control rate and survival rate of patients [192]. Despite these multimodal treatments improving outcomes, the survival rate remains low. As a result, researchers have extensively explored the combined use of multiple interventional techniques and nanotechnology, aiming to break through the limitations of monotherapy and traditional combination therapies.

The pancreatic cancer microenvironment typically features low pH, high levels of reducing substances (such as GSH) and a hypoxic environment, all of which make it difficult for CDT alone to achieve satisfactory therapeutic outcomes. Therefore, combining CDT with other therapies has emerged as a highly promising solution [193]. The integration of CDT with other treatments can produce significant synergistic effects, not only markedly enhancing the tumor-killing efficacy of CDT but also effectively reducing the side effects associated with CDT agents. For example, combining CDT with PDT or SDT is an effective strategy to increase the concentration and activity of ROS. The low-activity ROS generated by these two therapies can serve as substrates for CDT, further generating highly toxic hydroxyl radicals (•OH) through the Fenton reaction, thereby maximizing the advantages of both therapies [194, 195]. Additionally, increasing the temperature at the tumor site is also an effective method to enhance the efficacy of CDT.

PTT alone is limited by its restricted light penetration depth and issues related to thermal tolerance and thermal damage, making it challenging to effectively reach deep-seated tumors and completely eradicate cancer cells. Therefore, combining PTT with other therapeutic modalities has become a highly promising solution, aimed at overcoming these limitations and significantly enhancing the therapeutic efficacy [196]. Xue *et al*. developed an injectable hydrogel that integrates the near-infrared photothermal agent biliverdin (BV) and the immunomodulatory peptide thymopentin (TP5) for localized pancreatic cancer therapy. This hydrogel efficiently ablates local tumors via PTT and activates an immune response by releasing tumor-associated antigens (TAAs), thereby transforming the immunologically “cold” tumor microenvironment into a “hot” one. Meanwhile, TP5 further enhances the systemic immune response by regulating T-cell functions, thus, inhibiting tumor recurrence and metastasis. In addition, photothermal therapy alleviates tumor hypoxia by increasing the number of tumor blood vessels and dilating some of them, further optimizing the efficacy of immunotherapy [197]. Furthermore, the thermal effects of PTT can enhance the permeability of cell membranes, facilitating the entry of gene therapy vectors into tumor cells and thereby improving the efficiency of gene editing or gene silencing. This combinatorial therapeutic strategy not only enhances therapeutic efficacy but may also mitigate treatment-related side effects, as it allows for a reduction in drug dosage while simultaneously increasing the local concentration of the drug at the tumor site [198]. For instance, Wang *et al*. described a gene delivery nanomaterial composed of reduced graphene oxide (rGO) combined with gold nanostars (AuNS) and coated with a positively charged lipid bilayer. This design exhibits high photothermal conversion efficiency due to the presence of rGO@AuNS. The lipid bilayer not only enhances biocompatibility but also protects the genetic material from degradation by cellular enzymes, thereby enabling the potential for effective gene therapy. KrasI plasmids are delivered to the nucleus of pancreatic cancer cells via rGADA, where they are transcribed into short hairpin RNA (shRNA) and subsequently processed into siRNA. The siRNA then binds to mutant K-Ras mRNA, resulting in cell apoptosis and the inhibition of tumor metastasis. The highest tumor growth inhibition (TGI) of 98.5% was observed in the gene/photothermal treatment group [199].

**Figure 11.**
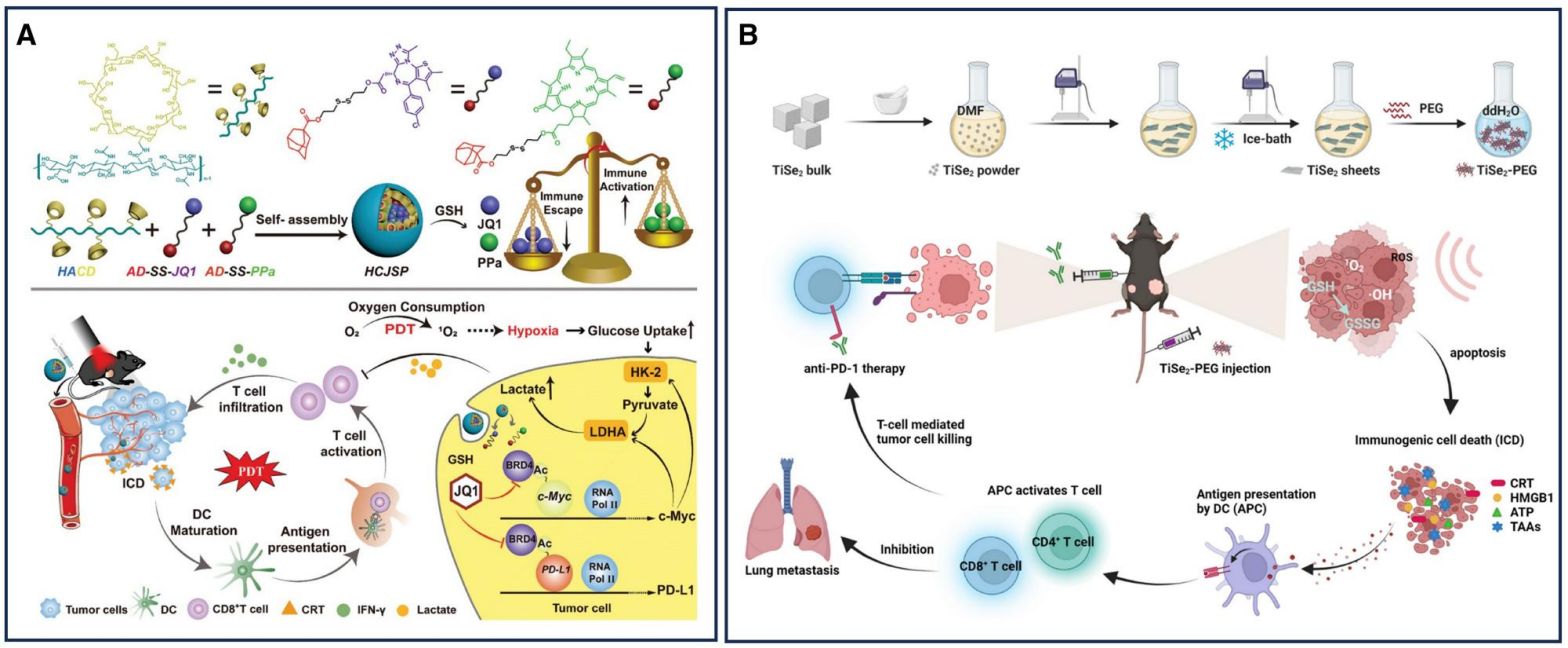
Nanomaterial-assisted synergistic therapy of pancreatic cancer. (**A**) PPa-mediated PDT for enhancing tumor cell immunogenicity and overcoming adaptive immune resistance. Reproduced with permission [201]. Copyright 2021, Wiley‐VCH GmbH. (**B**) TiSe2-mediated SDT and anti-PD-1 for the treatment of pancreatic cancer and inhibition of its growth and metastasis. Reproduced with permission [202]. Copyright 2022, Springer Nature.

PDT has the potential to induce immunogenic cell death in tumor cells, which may contribute to the release of tumor antigens and the subsequent activation of antitumor immune responses. However, the tumor microenvironment of pancreatic cancer typically exhibits immunosuppressive characteristics, including a high prevalence of regulatory T cells (Tregs) and myeloid-derived suppressor cells (MDSCs). These cells can impede the immune response, leading to immune evasion [200]. Yu *et al*. developed a supramolecular prodrug nanomaterial that delivers a photosensitizer as well as a prodrug carrying the bromodomain-containing protein 4 inhibitor (BRD4i) JQ1 for pancreatic cancer photo-immunotherapy. The nanomaterial was complexed with cyclodextrin-grafted hyaluronic acid (HA-CD), amantadine-coupled pyrophosphate dimer a (PPa) and the host-guest JQ1. Among the components, hyaluronic acid facilitates active targeting of pancreatic cancer, PPa generates ROS under NIR laser irradiation following internalization into tumor cells and enhances immunogenicity, while JQ1 counteracts PDT-mediated immune evasion by inhibiting the expression of c-Myc and PD-L1, key regulators of tumor glycolysis and immune evasion (Figure 11A) [201].

Moreover, SDT can be employed in conjunction with immune checkpoint inhibitors to stimulate antitumor immune responses through the induction of tumor immunogenic cell death (ICD). To illustrate, the construction of cavitation-assisted endoplasmic reticulum-targeted acoustic kinetic nanodroplets has the potential to enhance the accumulation and penetration of the loaded acoustic sensitizer TiSe_2_ within the tumor (Figure 11B). Upon exposure to noninvasive US irradiation, these TiSe_2_ nanodroplets generate substantial levels of ROS, irrespective of the oxygenation status of the surrounding environment. This results in the maturation and cytokine secretion of dendritic cells (DCs). This activation process promotes the infiltration of CD8^+^ T cells into the tumor while simultaneously reducing the number of regulatory T cells (Tregs), thereby enhancing the systemic immune response and facilitating the elimination of pancreatic tumors [202].

## Nanomaterials in translational and clinical medicine

In recent years, nanotechnology, as a paragon of interdisciplinary research, has achieved revolutionary progress in the field of precision theranostics through the deep integration of materials science and biomedicine. The application of nanomaterials is gradually transitioning from the laboratory to clinical practice [33]. Currently, in the field of medical imaging diagnostics, multimodal imaging probe technology based on nanomaterials has significantly optimized the early detection rate and lesion localization accuracy of pancreatic cancer by synergistically integrating multiple imaging modalities. Taking Ga-based nanomaterials as an example, they selectively accumulate in the lesion area by enhancing the EPR effect, thereby improving the contrast resolution of clinical MRI. This mechanism has been widely applied in the radiological assessment of pancreatic cancer [50]. However, the limitations of traditional nontargeted nanoprobes have prompted researchers to focus on the development of specific molecular diagnostic tools. Recent breakthroughs indicate that cetuximab-IRDye800 conjugates targeting the epidermal growth factor receptor (EGFR) have entered the clinical trial stage for fluorescence-guided surgery (FGS) in pancreatic cancer, providing a noninvasive monitoring method for tumor molecular subtyping [203]. In addition, the integration of nanomaterials and spectroscopy has further expanded the boundaries of intraoperative diagnosis. For example, gold nanoparticles (AuNPs) leverage their surface plasmon resonance properties in combination with surface-enhanced Raman spectroscopy (SERS) to significantly amplify the Raman signal intensity of biological tissues. Recent small-scale clinical studies have shown that this strategy can achieve high-resolution identification of microscopic metastases (<2 mm) in pancreatic cancer during surgery (with a spatial resolution of up to 50 μm), breaking through the sensitivity limitations of traditional intraoperative imaging. This provides a revolutionary technological support for precise surgical navigation and radical tumor resection [204]. More importantly, the concept of theranostics in nanotechnology has given rise to several nanoplatforms that have entered the clinical stage for pancreatic cancer. For example, liposome-encapsulated irinotecan (Onivyde) extends the drug’s half-life through long-circulating design and, in combination with the 5-FU regimen, increases the median survival of patients with metastatic pancreatic cancer to 11.1 months. It has currently been approved by the FDA as a first-line treatment option [205]. In addition, Abraxane (paclitaxel-albumin complex) based on albumin nanoparticles significantly enhances drug accumulation in pancreatic tumors through gp60 receptor-mediated trans-endothelial transport. Its combination with gemcitabine has been incorporated into the NCCN guidelines as the standard therapy for locally advanced pancreatic cancer [206]. However, the design of nanomaterials has primarily focused on the macroscopic therapeutic effects on tumors, and there are still significant limitations in overcoming the pancreatic cancer tumor microenvironment and innovating in diversified diagnosis and treatment [32]. Scientists are currently committed to developing cutting-edge nanomaterials in the heterogeneous microenvironment of pancreatic cancer to address the challenges of precise diagnosis and targeted therapy for pancreatic cancer.

## Summary and outlook

Despite decades of advances, pancreatic cancer is widely recognized as one of the most challenging malignancies to diagnose and treat, largely due to its asymptomatic progression, late-stage detection and the presence of a dense stromal microenvironment that significantly impairs therapeutic efficacy. Nanomaterials have emerged as highly promising platforms in the field of theranostics, offering the ability to integrate diagnostic and therapeutic functionalities within a single system. Recent advancements in nanomaterial design, including metallic nanoparticles, lipid-based carriers, polymeric nanoparticles and carbon-based nanostructures, have markedly enhanced both imaging sensitivity and therapeutic delivery. These materials have demonstrated exceptional potential in improving diagnostic imaging modalities such as MRI, CT and PET by facilitating tumor visualization through the use of targeted contrast agents. Concurrently, nanomaterials enable precise drug delivery, effectively traverse biological barriers and support combination therapies such as chemo-photothermal and immunotherapy, which have exhibited enhanced efficacy in preclinical studies. Nevertheless, despite these significant advancements, challenges related to biocompatibility, toxicity, large-scale production and regulatory approval continue to impede the clinical translation of nanomaterial-assisted theranostics.

The future of nanomaterial-assisted theranostics for pancreatic cancer presents significant potential, with numerous innovative advancements anticipated. The development of smart nanomaterials responsive to tumor-specific stimuli, such as pH variations, enzymatic activity and hypoxic conditions, is expected to enable highly precise and controlled drug release. Progress in hybrid nanomaterials that integrate multiple imaging and therapeutic modalities will further enhance their multifunctional capabilities, facilitating real-time monitoring of therapeutic efficacy. Additionally, the integration of artificial intelligence (AI) and machine learning into nanomedicine holds promise for optimizing nanoparticle design, predicting patient-specific therapeutic responses and enabling personalized treatment strategies. Addressing existing challenges, such as enhancing biocompatibility, ensuring safety and achieving scalability for large-scale production, will require concerted interdisciplinary collaboration. With continued research and innovation, nanomaterial-based precision diagnostics and therapies have the potential to revolutionize pancreatic cancer management, significantly reducing its high mortality rate and improving clinical outcomes.
